# National respiratory syncytial virus acute respiratory infection burden of disease estimate in Mexican children under 2 years

**DOI:** 10.3389/fped.2026.1880884

**Published:** 2026-09-09

**Authors:** Rosa María Wong-Chew, Cristina Gutiérrez-Delgado, Norberto F. Hernández-Llanes, Daniel E. Noyola, Carlos F Reyes-Hamburguer, Patricia Guadalupe Cervantes-Powell, Alejandra López-Neri

**Affiliations:** 1 Head of the Subdivision of Clinical Research, Facultad de Medicina, Universidad Nacional Autónoma de México (UNAM), Mexico, Mexico; 2 Department of Actuarial Sciences, Faculty of Sciences, Universidad Nacional Autónoma de México (UNAM), Mexico, Mexico; 3 El Poder del Consumidor A.C., Mexico, Mexico; 4 Research Center in Health Sciences and Biomedicine (CICSaB), Universidad Autónoma de San Luis Potosí, San Luis Potosí, Mexico; 5 Medical RSV Lead Latam, Sanofi LaTam, Bogota, Colombia; 6 Medical Lead Mexico, Sanofi Mexico, Mexico, Mexico; 7 RSV Medical Advisor Mexico, Sanofi Mexico, Mexico, Mexico

**Keywords:** acute respiratory infections, children, hospitalization, incidence, mortality, outpatient and emergency department, population based studies, respiratory syncytial virus

## Abstract

**Background:**

National population-based respiratory syncytial virus (RSV) direct burden-of-disease estimates on outpatient, emergency room (ER) and hospitalization settings are scarce among Mexican children. These estimates are key to inform the development of preventive public policies and to strengthen seasonal clinical planning in Mexico.

**Methods:**

A systematic review of literature allowed estimating robust RSV-proportions in acute respiratory infection (ARI) and lower respiratory tract infection (LRTI) studies in Mexican children 0 to 23 months-old (<2yo) and length-of-stay, mortality rates, mechanical ventilation use, and seasonality patterns for RSV-attributed cases that could be scaled nationally. These results allowed the estimation of outpatient and ER visits, hospitalizations, total length-of-stay and deaths of RSV-related illnesses from national databases available in the public domain for the 2011–2023 period. Incidence rates were also calculated considering the official population projections for the period analyzed.

**Results:**

RSV-proportions accounted for 31.76% and 16.03% of outpatient visits in children 0 to 11 months-old (<1yo) and children 12 to 23 months-old (1yo) respectively; In <2yo RSV-proportions were 17.96% in ER and 20.25% (pneumonia) to 34.72% (bronchiolitis) in hospitalization. Mean length-of-stay and mortality were highest in preterm <1yo (7.04 days, 2.11%) followed by at-term <1yo (5.77 days; 1.4%) and 1yo (4.77; 0.87%). Mechanical ventilation followed a similar trend (12.24%, 5.77% and 2.03%). Seasonally (96% autumn-winter), RSV caused in <2yo ∼772,450 outpatient visits, ∼185,326 ER visits, and ∼26,873 hospitalizations. Annual rates per 1,000 children were ∼248 and ∼107 in <1yo and 1yo respectively in outpatient settings; ∼55, ∼30 in ER settings and ∼32 in preterm <1yo, ∼9 at-term <1yo and ∼4 in 1yo in hospitalization settings.

**Conclusions:**

RSV burden-of-disease was seasonal, imposing a major burden on healthcare facilities during autumn-winter. Children <1yo were the most affected in the three clinical settings analyzed. These results can be used to calculate the financial RSV burden-of-disease as well as to inform the development of preventive policies and to strengthen seasonal clinical planning in Mexico.

## 1 Introduction

Respiratory syncytial virus (RSV) is a leading cause of acute respiratory infection (ARI) in children 0 to 23 months-old (<2yo). Its natural history ranges from common cold and otitis media to bronchiolitis, pneumonia, and even death if timely medical care is not provided (1). Most RSV-infections are treated in outpatient settings leaving the most severe cases, mainly bronchiolitis and pneumonia, to be treated in hospital settings. Preterm birth or having certain medical conditions, such as congenital heart disease or bronchopulmonary dysplasia, are known risk factors for serious complications (2, 3). However, most cases of severe RSV-infections are seen in previously healthy children born at term (4). Several studies have reported estimates of RSV-hospitalization rates in specific countries, finding consistent results in terms of seasonality and most affected age groups (5, 6). However, estimates of hospitalization rates using specific RSV International Classification of Diseases, 10th Revision (ICD-10) codes extracted from administrative data available in public domain were notably lower than those reported using other methods (7, 8). These findings documented that application of laboratory tests to all ARI hospitalizations to identify RSV is not a common clinical practice. Thus, studies based on administrative data used a wider group of ARI ICD-10 codes (4).

In Mexico, several studies have been conducted to determine RSV burden-of-disease in terms of morbidity and mortality in hospital settings. These used epidemiological surveillance, hospital cohort follow-up, population-based methods and mathematical models for the estimation of RSV hospitalizations (2, 9–13). However, there are no RSV hospitalization estimates with national representativeness based upon public administrative data. Likewise, published studies on RSV burden-of-disease in outpatient or emergency room (ER) settings with national representativeness are scarce (14, 15). These estimates are key to inform the development of robust preventive public policies in Mexico.

Obtaining national estimates of RSV disease burden in children is an operational requirement to translate virological and clinical evidence into public health decision and hospital management. National estimates allow for quantifying the magnitude of the problem in terms of expected demand, identifying high-risk groups, characterizing local seasonality, and establishing a baseline to evaluate trends and the impact of preventive interventions. This is particularly relevant at a stage when effective preventive tools have emerged (monoclonal antibodies and maternal vaccines), whose population-level effect must be measured against a reliable and historically comparable benchmark.

Furthermore, the absence of reliable national estimates limits the allocation of hospital beds and critical care capacity (general pediatrics, high-flow oxygen, PICU), as it prevents accurately anticipating the expected healthcare pressure and the margin needed to absorb peaks. This increases the risk of saturation, rescheduling of elective activities, and unwanted variability in admission and discharge decisions. Lastly, the lack of data hinders the development of preventive policies: without quantifying disease burden and distribution (by age, region, social vulnerability, and comorbidities), it is difficult to define coverage goals, prioritize populations, select strategies (maternal vaccination vs. infant immunization), budget for procurement, or evaluate cost-effectiveness and equity impact.

## 2 Materials and methods

**Figure 1:**
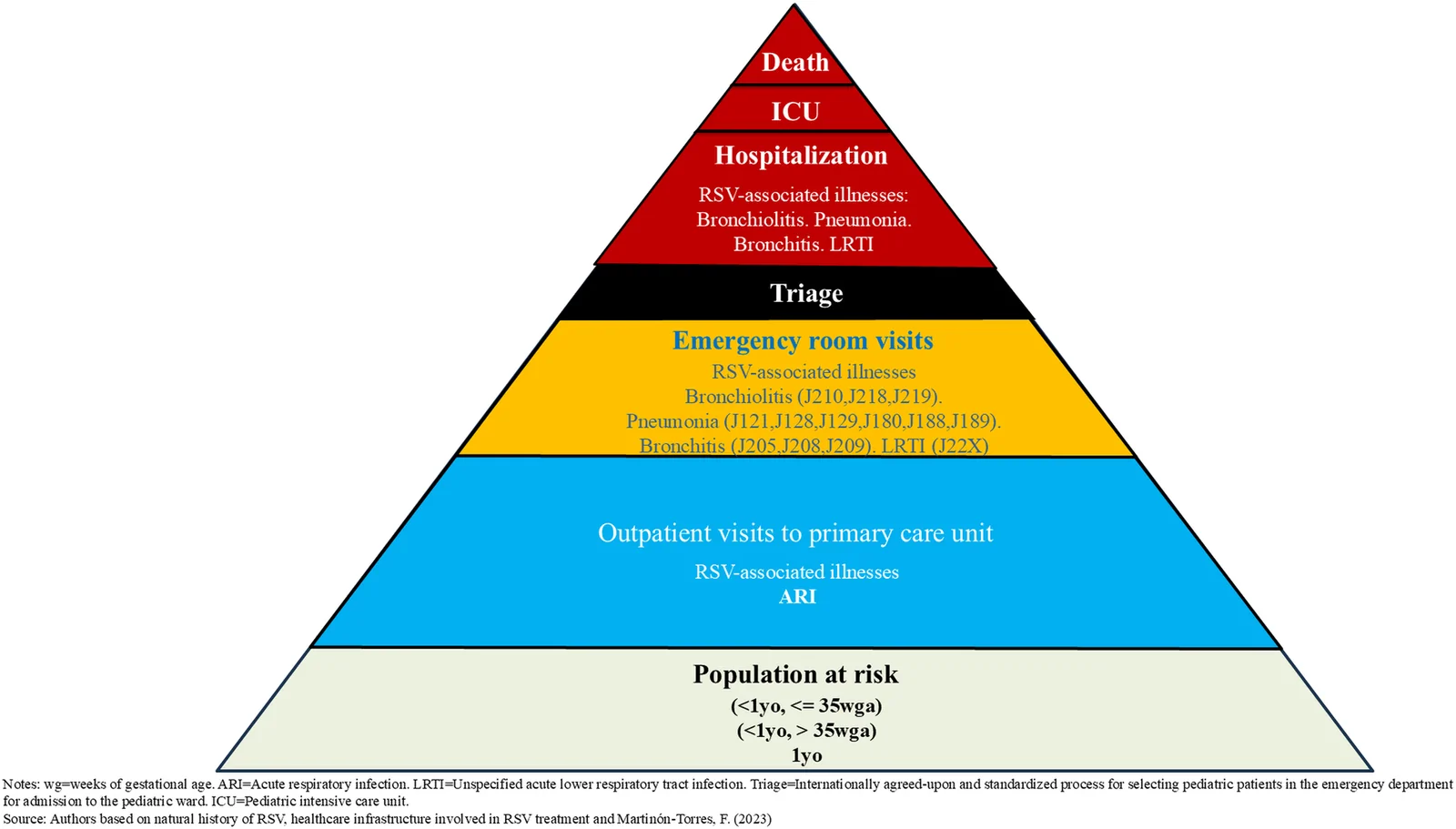
Population-based RSV burden-of-disease framework for analysis. wga, weeks of gestational age; ARI, Acute respiratory infection; LRTI, Unspecified acute lower respiratory tract infection; Triage, Internationally agreed-upon and standardized process for selecting pediatric patients in the ER for admission to hospitalization in the pediatric ward; ICU, Pediatric intensive care unit. Source: Authors based on natural history of RSV, healthcare infrastructure involved in RSV treatment, Martinón-Torres et al. (2023) (4) and Mata-Moreno et al. (2024) (11).

Population-based RSV burden-of-disease studies require calculating cases of RSV-infections among a population at risk at various points of care throughout the natural history of disease (see Figure 1) (16). In this study we estimated RSV burden-of-disease in three healthcare settings (outpatients, ER, and hospitalizations). Children <2yo were the population at risk as this group was reported as the most affected in published studies (2, 9–13). Segmentation in children newborn to 11 months (<1yo) and 12 to 23 months (1yo) was used for the analysis of outpatient and ER. An additional segmentation per week of gestational age (wga) was applied to hospitalized <1yo resulting in estimates for those born at ≤35wga (preterm) and those born at >35wga (at term).

### 2.1 Databases sources

Databases of outpatient, ER and hospital activity provided in the healthcare units of the public, social security and private subsystems at national level available in the *Sistema Nacional de Información Básica en Materia de Salud* and the *Instituto Nacional de Estadística y Geografía* (INEGI) platforms were used. (See Supplementary Digital Content 1 Table for further details) (17–20). The period of study was 2011 to 2023. However, 2020–2023 data were analyzed separately as the epidemiological profile and reporting priorities changed due to the COVID-19 pandemic. The data mining was performed using R version 4.2.2. As reporting to the information systems is not legally binding, a proportion of the healthcare units did not report medical activity. An extrapolation by proportionality based on the annual inventory of operative healthcare facilities for each healthcare subsystem analyzed was performed to solve the annual gap of no report (21). (See Supplementary Digital Content 2 Text and Tables for further details on this extrapolation process). Selected ICD-10 codes for ER and hospitalization databases followed those used by Martinón-Torres et al. (2023) (4) as very few hospitalizations were registered with RSV ICD-10 codes.

### 2.2 Systematic review of RSV studies

A systematic review of literature for RSV-infections studies in Mexican children <2yo was developed following the methodology of the Cochrane Collaboration and Joanna Briggs Institute (22, 23). Following the guidelines of the Preferred Reporting Items for Systematic Review and Meta-Analysis Protocols (PRISMA-P), the SR protocol was registered in the International Prospective Register of Systematic Reviews (PROSPERO) under number CRD42024617732 (24). The primary objective of this review was to analyze studies related to clinical settings in Mexico that reported RSV-percentages among ARI and LRTI. This approach ensured that results could be scaled nationally using standard epidemiological and statistical methods (25–27). Findings from 36 out of 4,110 published papers and grey literature documents identified allowed estimating robust RSV-proportions and secondary outcomes (length-of-stay, mortality rates, percentage of ICU admissions, percentage of MV use) for the three healthcare settings analyzed. Findings also permitted the development of three scenarios (Mean, minimum-Min, maximum-Max) for the different RSV-proportions that were applied to estimate RSV cases and rates. Additionally, two statistical analyses were performed. One to data collected from different RSV studies to obtain monthly distribution of RSV-LRTI (2, 9–11, 13). Other to ER databases to obtain cases distribution of ER discharge categories. Data sources used in both analyses allowed that results were representative at national level.

### 2.3 Estimation of RSV cases

The estimated number of RSV-cases from the databases analyzed were obtained by applying the RSV-proportions to the selected ARI cases. (See Supplementary Digital Content 2 text and equation A for further details on this estimation process). Once the estimated numbers of RSV-cases in the outpatient and ER databases were obtained, an extrapolation was applied to obtain the total estimated number of RSV-cases. This extrapolation was based on the proportion of operative healthcare facilities (primary care or hospitals) from social security and private subsystems in comparison with the proportion of public subsystem healthcare settings. (See Supplementary Digital Content 3. Text for further details). This extrapolation was not required in hospitalization databases as there was a dataset for each subsystem.

The secondary outcomes from the systematic review were applied to the total number of RSV hospitalizations to estimate number of cases using mechanical ventilation as proxy of pediatric intensive-care-unit admission given the absence of this last variable on the Databases, total days of hospital stay and deaths. (See Supplementary Digital Content 4 Table for these results). Additionally, an adjustment to the monthly distribution of total RSV-cases in the three healthcare settings analyzed was performed by using the monthly distribution of RSV in LRTI.

National and state population-based annual incidence rates per 1,000 children <2yo were calculated by using standard epidemiological methods (25). Mid-year population per age projected by the National Population Council (CONAPO) was used for the estimations (28). This population was adjusted to reflect the actual difference between the projection and the birth registry for the period 2011–2019 (28, 29). (See Supplementary Digital Content 5. Text and Table for further details on the adjustment procedure and the at-risk population results). An additional segmentation by wga was applied to infants <1yo resulting in two groups: those born preterm and those born at term. The percentage for the wga segmentation was the one reported in the birth registry annually (28). This segmentation was used for the hospitalization rates only as there was no published data on preterm children with RSV-infections in outpatient nor ER visits.

## 3 Results

### 3.1 RSV proportions from the systematic review

Table 1 presents the results of the systematic review. The percentage of RSV-cases in outpatient visits almost doubled the percentage of RSV-cases in ER and hospitalization. In hospitalized patients, pneumonia was the RSV-associated illness with the longest stay (6.5 days) and highest mortality rates (1.4%). RSV-proportions of pediatric intensive care unit admission per age follow similar patterns than the RSV-proportion of mechanical ventilation (very high for <1yo preterm, high for <1yo at term, low for 1yo). These results allowed the use of mechanical ventilation as proxy of pediatric intensive care unit admission in absence of the latter variable in the databases. Of note, although mechanical ventilation use was less frequent than pediatric intensive care unit admission, the former accounts for the most severe RSV-cases. Preterm infants <1yo showed the highest use of mechanical ventilation (12%), mortality (2.11%) and longest hospital stay (7 days). Seasonality analysis showed that ∼97.5% of RSV-infections occurred during autumn-winter seasons (from October to March). Most RSV cases were discharged home, approximately 20% required hospitalization, and less than 0.1% died although mortality increased in 2020 and 2021 (See Supplementary Digital Content 6 for further details on seasonality and discharge analyses).

**Table 1:** RSV-Proportions and secondary results from systematic review.

RSV-proportions	Mean	Min	Max
% of RSV in total outpatient visits for ARI <1yo	31.76	26.10	38.01
% of RSV in total outpatient visits for ARI 1yo	16.03	11.89	21.27
% of RSV in emergency visits for LRTI <2yo	17.96	5.25	46.41
Hospitalization for <2yo			
% of RSV in total pneumonias	20.25	7.86	43.04
% of RSV in total bronchitis/1	22.22		
% of RSV in total bronchiolitis	34.72	26.88	43.50
% of RSV in total LRTI	33.37	28.02	39.18
Secondary outcomes for RSV proportions in hospitalization <2yo			
Length of stay (days)	Mean	StanDev	
RSV-associated LRTI	5.83	5.89	
RSV-associated pneumonia	6.51	5.35	
RSV-associated bronchitis	NA	NA	
RSV-associated bronchiolitis	4.82	4.45	
Mortality rate (%) < 2yo	Mean	Min	Max
RSV-associated LRTI	1.14	0.46	2.8
RSV-associated pneumonia	1.43	0.54	3.74
RSV-associated bronchitis	NA	NA	NA
RSV-associated bronchiolitis	0	0	0
Secondary outcomes for RSV proportions in LRTIs in hospitalization			
% Intensive Care Unit Admission	Mean	Min	Max
<1yo	7.1	3.03	15.75
1yo	5.04	0.97	22.32
<1yo, preterm	13.17	9.69	17.65
% Mechanical Ventilation	Mean	Min	Max
<1yo	5.65	4.49	7.08
1yo	2.03	0.91	4.44
<1yo, preterm	12.24	8.70	16.97
Length of stay	Mean	StanDev	
<1yo	5.77	5.76	
1yo	4.77	4.32	
<1yo, preterm	7.04	5.62	
Mortality rate	Mean	Min	Max
<1yo	1.4	0.43	4.46
1yo	0.87	0.28	2.66
<1yo, preterm	2.11	0.95	4.61

/1. Data from a single reference. Mean: Mean weighted by sample size. Min/Max: Minimum/Maximum value identified in the systematic review. yo: year old. LRTI: lower respiratory tract infection. wga: weeks of gestational age at birth. StandDev: Standard deviation. Source: Burden of acute respiratory infections caused by respiratory syncytial virus in children under 24 months old in Mexico: a systematic review. Full SR report is available at the request to authors.

### 3.2 Estimated RSV cases in outpatient, ER and hospitalized settings

**Figure 2:**
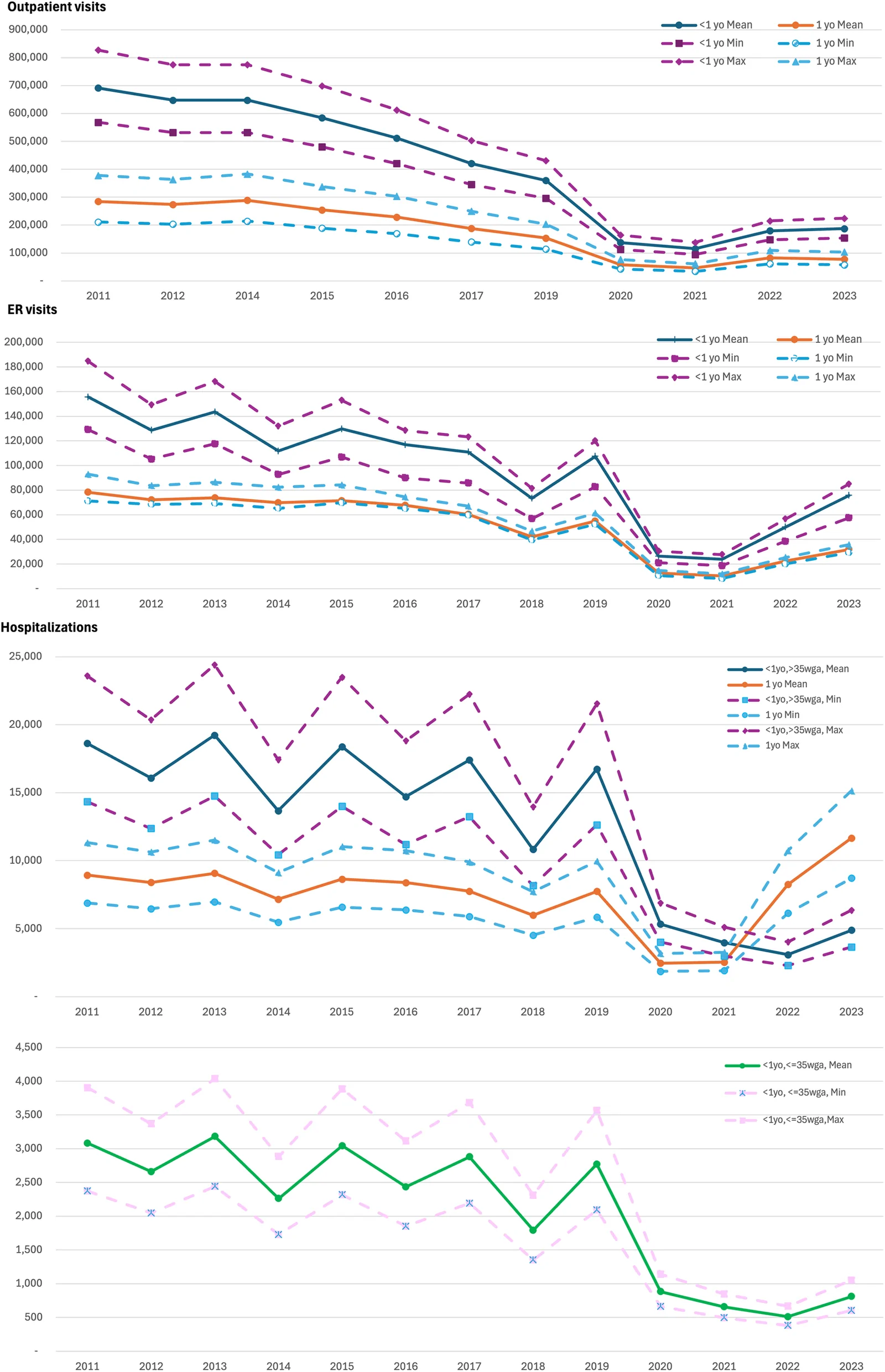
Estimated RSV cases in outpatient visits, ER visits and hospitalizations. yo, years old; wge, weeks of gestational age at birth; ER, emergency room. Source: Authors estimates based upon (ProgNiñoSano, SUMHo, SAEH, SEHS, SESEP, RegCLUES) and RSV proportion of cases.

Results in each healthcare setting analyzed were reported for the three scenarios generated by the RSV-proportions and identified as follows: Mean Scenario (MS), Min Scenario (MiS), Max Scenario (MaS). Figure 2 presents total estimated RSV-cases for each year and age group for the three healthcare settings analyzed. During the pre-pandemic period (2011–2019), the mean yearly estimated number of RSV-infections in outpatient, ER, and hospitalizations in <2yo in the MS were 772,450, 185,326, and 26,873, respectively. An annual mean of 539,463-MS (443.325-MiS;645,623-MaS) RSV outpatient visits were estimated among <1yo. In contrast, annual mean outpatient visits among 1yo reached 232,987-MS (172,815-MiS;309,148-MaS), only 43%-MS (39%-MiS;48%-MaS) compared with the <1yo figure. As for RSV ER visits, an annual mean of 119,757-MS (96,301-MiS;137,776-MaS) was estimated for <1yo. In comparison, among 1yo the annual mean reached 65,569-MS (65,024-MiS;75,415-MaS) representing 55%-MS (68%-MiS;55%-MaS) of the figures for children <1yo. Annual mean hospitalizations among preterm <1yo were estimated at 2,678-MS (2,044-MiS;3,417-MaS) comprising 14% of hospitalizations in <1yo, while annual mean hospitalizations in at-term <1yo were 16,184-MS (12,351-MiS;20,646-MaS), comprising 86% of all hospitalizations in <1yo. Meanwhile, estimated hospitalizations among 1yo accounted for 42%-MS (49%-MiS;50%-MaS) of those figures for <1yo at-term. Cases for 2020–2023 showed a different pattern linked to the COVID-19 pandemic that changed the epidemiological profile and the reporting priorities. Thus, results were not comparable with previous years analyzed and their interpretation and use should be carried out with caution. It is worth noting that, compared with the 2019 figures, the reduction in the estimated number of cases for outpatient visits was 62% in 2020, 69% in 2021, 22% in 2022, and 48% in 2023; for ER visits 76%, 79%, 55%, and 34%, respectively; and for hospitalizations 68%, 74%, 57%, 36%, respectively. (See Supplementary Digital Content 7 for actual numbers). Secondary outcomes in hospital settings showed that around 1,420 < 2yo used MV during hospitalization each year. The yearly mean number of deaths associated with RSV amounted to 356, and a total of 152,211 hospital days were generated each year. Overall, most of these cases occurred among at-term <1yo. (See Supplementary Digital Content 4 Table for further details).

### 3.3 Seasonal distribution of estimated RSV cases

**Figure 3:**
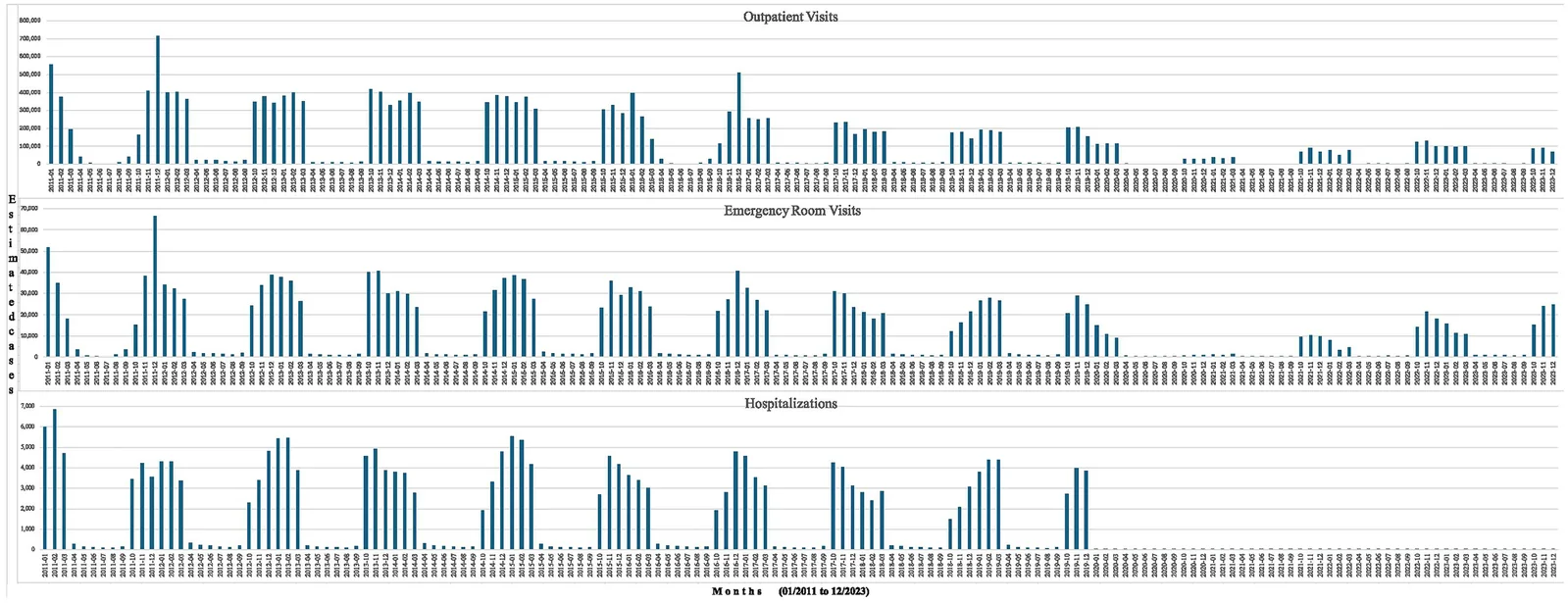
Monthly distribution of estimated RSV-related illnesses per healthcare setting. 2011-2023. No data on monthly distribution of cases for hospitalization databases in 2020-2023. Source: Authors calculations based upon (ProgNinSan, SUMHo, SAEH, SEHS, SESEPE) and data from Figure 2.

Figure 3 shows the seasonal distribution of the estimated RSV-cases in each of the three healthcare settings analyzed. A mean 96%-MS (95%-MiS,97%-MaS) of RSV-related illnesses were registered during autumn-winter, reaching peaks in December-January. Worth noting, the hospitalization databases for the period 2020–2023 provided only total figures, without indicating the number of admissions that occurred per month. Thus, it was not possible to estimate or extrapolate the monthly distribution for this period. As for outpatient and ER visits, the monthly distribution during 2020 and 2021 was highly concentrated in January and February 2020, with no peak in October-December of that year nor in January-March 2021, reflecting the epidemiologic change generated by the COVID-19 pandemic. The monthly distribution returned to pre-pandemic trends as of October 2021.

### 3.4 National population-based estimated incidence rates of RSV

**Figure 4:**
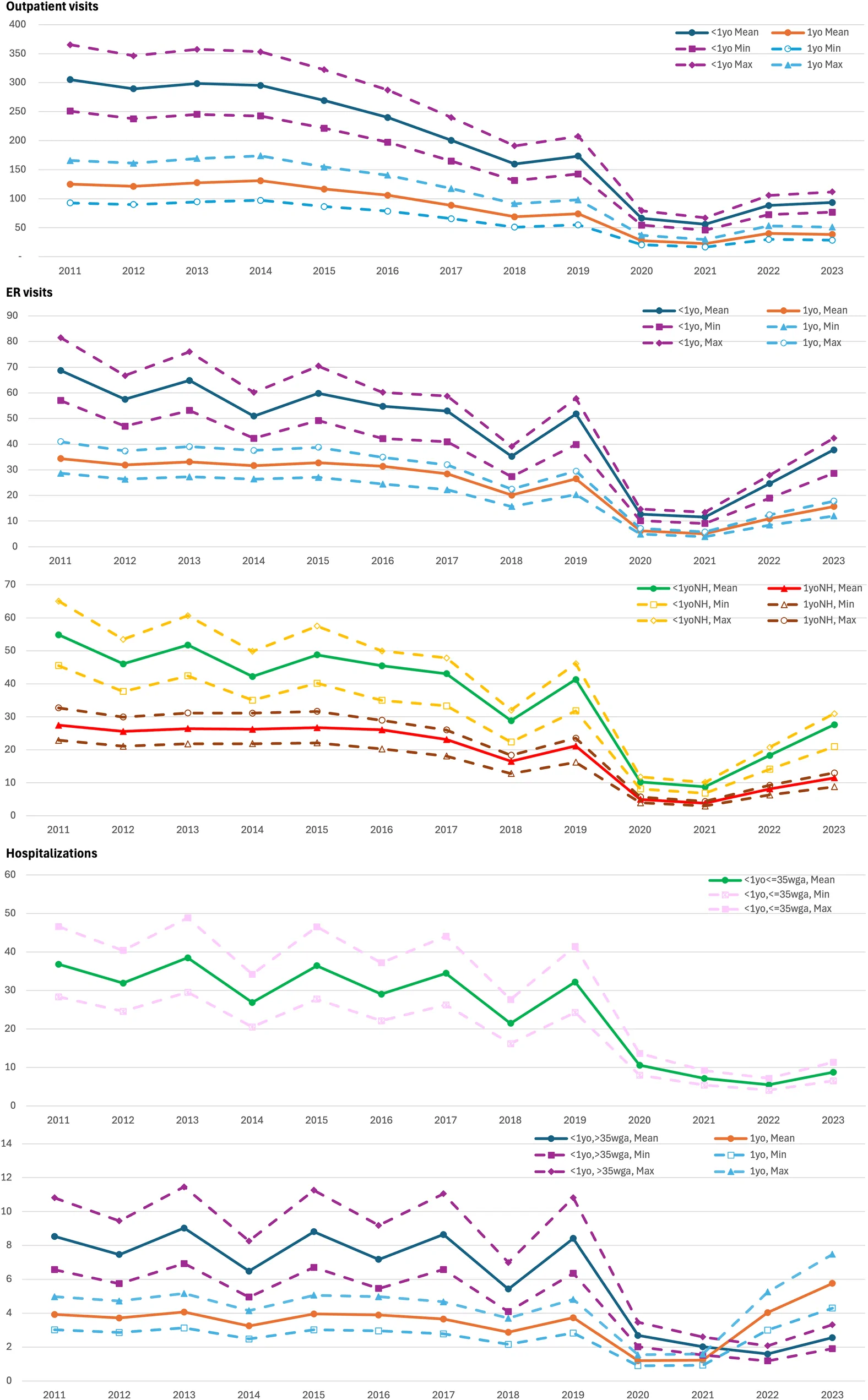
Incidence rates of RSV-cases per 1,000 children, age group and healthcare setting. yo, years old; ER, emergency room; NH, without hospitalizations; wga, weeks of gestational age at birth. Source: Authors estimates based upon information from population at risk and estimated number of RSV-cases.

Figure 4 shows estimated national population-based incidence rates for the three healthcare settings analyzed. Rates were expressed in numbers of cases per 1,000 children of each age group analyzed. A national mean of 2.1 million <1yo (83,491 preterm) and 2.2 million 1yo were at risk of developing RSV-related illnesses each year. For ER visits, rates were calculated with and without the proportion of children that passed the *triage* to be hospitalized. Rates without hospitalizations prevent an overestimation of cases that were also included in the hospitalization databases. Meanwhile rates with hospitalizations allow to quantify the total number of cases that reach ER settings. Hospitalization rates for infants <1yo were calculated for those preterm and at-term to allow full understanding of the role of prematurity as a risk factor for hospitalization. In 2011–2019 the annual mean rate of outpatient visits for <1yo was 248-MS (204-MiS;297-MaS) and for 1yo was 107-MS (79-MiS;141-MaS). Notice that the RSV-proportion of outpatient visits is the result of the meta-analysis reported in the Systematic Review, which included three studies conducted in Mexico: Manjarrez et al., 2003 (30); Cabello et al., 2006 (31); and Wong et al., 2015 (15). As for ER visits, mean rates in <1yo were 55-MS (44-MiS;63-MaS) with hospitalizations and 45-MS (36-MiS;51-MaS) without hospitalizations. Mean ER rate in 1yo was 30-MS (24-MiS;35-MaS) with hospitalizations and 24-MS (20-MiS;28-MaS) without hospitalizations. Mean hospitalization rates were 8-MS (6-MiS;10-MaS) and 32-MS (24-MiS;41-MaS) for at-term and preterm <1yo respectively, while in 1yo was 4-AS (3-MiS;5-MaS). These estimates reflected the fact that the preterm population is at higher risk of developing RSV-infections but only represents a small proportion of the overall burden. (See Supplementary Digital Content 8 for actual numbers).

Given the significative variability of cases reported per state, an estimate of the incidence rates at subnational level was developed. Results are presented in Supplementary Digital Content 8. Main findings document that the largest incidence rate is around five times the smallest in outpatient settings, while this difference reaches up to 357 times in ER settings and up to 8 times in hospitalization.

## 4 Discussion

Results from this study provide for the first time national and subnational population-based estimate of the RSV burden-of-disease in Mexican children <2yo for the period 2011–2023. The study analyzed public data in three key healthcare settings using standard epidemiological and statistical methods and robust RSV-proportions and secondary outcomes generated from a systematic review.

A mean of 2.1 million children <1yo (83,491 preterm) and 2.2 million 1yo were at risk of developing RSV-related illnesses each year. Following the natural history of RSV the estimated number of outpatient visits was significantly higher than the estimated number of ER visits and hospitalizations (772,450 vs. 185,326 vs. 26,873 annual mean). In hospital settings an estimated annual mean of 1,420 children <2yo used MV while an estimated of 356 deaths occurred, generating an estimated of 152,211 hospital days, being children <1yo at term the biggest contributors. On average, 96% of RSV-infections in the three healthcare settings were registered during autumn-winter, reaching peaks in December-January and concentrating in children <1yo. Estimated hospitalizations among preterm <1yo represented 17% of estimated <1yo hospitalizations. This finding underlines the need to protect all children <1yo as strategies targeting only preterm would address a small proportion of the overall burden of RSV.

On average, estimated outpatient visits rates were 3.9 times those of ER visits and 28.8 times the ones of hospitalization. It is worth mentioning the significant gap between ER and hospitalization rates, averaging 5.8 times. This gap and the fact that hospitalization rates for <1yo at term and 1yo were significantly lower than those reported in studies specifically focused on RSV-case detection (8–11, 14, 15), could be partially explained by the application of the “*triage*” in ER to determine which cases are hospitalized, especially during the winter peak of LRTI. Triage in ER prioritizes care based on clinical severity. Therefore, patients with significant respiratory failure are priority for hospitalization, while less critically ill patients are treated in ER. According to national clinical practice guidelines, children with moderate to severe pneumonia are hospitalized based on several factors, including respiratory distress and hypoxemia (oxygen saturation <92%) (31). Of note, even if the stay in the ER is longer than one day, it is not recorded as a hospitalization. Findings on the RSV-associated emergency discharge categories (see Supplementary Digital Content 6 Table SDCT6.2) indicated that after *triage* application the annual hospitalization was on average 21%.

Results were presented for period 2011–2019 separated from results for period 2020–2023 allowing an easy detection of epidemiological differences generated by the COVID-19 pandemic. Overall trends in 2011–2019 showed a light decreasing pattern in the numbers of outpatient visits, ER visits and hospitalizations that might be related to the decreasing projection of children <2yo estimated by CONAPO.

The high burden of RSV infections in young children is a worldwide concern (6). This has led to development of specific preventive interventions, including monoclonal antibodies (palivizumab, nirsevimab, and clesrovimab) and vaccines directed against this virus (32–35). Currently, palivizumab and nirsevimab are available in Mexico for administration in infants. RSV maternal immunization is also available for prevention of severe infections in young children and has recently been included in the National Immunization Program. In contrast, 2020–2023 trends showed a significant decrease as a direct result of the public policies implemented during the COVID-19 pandemic. The biggest decrements were registered in 2020–2021 while in 2022–2023 decrements were smaller although not enough to reach the pre-pandemic pattern.

## 5 Limitations and future research

Estimates for outpatient and ER visits as well as hospitalizations presented a decreasing pattern along the 2011–2019 period. While this pattern could be related to the decrease in the population at risk projected by CONAPO it could also be related to an increase in both the missing transcriptions of paper clinical records into electronic records and the partial report of clinical activity within the same healthcare facility as can be seen in incidence rates for states with big number of <2yo at risk but little reported RSV-infections (see Supplementary Digital Content 8). In addition, outpatient databases did not include information on the actual number of outpatient facilities reporting, preventing the application of the extrapolation process to solve the actual no report that could be solved for ER and hospitalization. Another limitation on the extrapolation of cases is the assumption that reporting and non-reporting facilities are comparable. While the assumption was used considering the national inventory of facilities, the assumption may not hold for a subnational analysis as facilities differ in patient volume, disease severity, diagnostic practices, geographic distribution and health access. Additional limitations of this study were related to the RSV-proportions. The systematic review for RSV-proportions in ER settings only identified data for the age group <2yo and for ARI even though the ER databases included individual ages and registered the ER visit by ICD-10 codes. And although the RSV-proportions allowed for the development of three scenarios useful for a descriptive analysis they might not fully capture uncertainty in counting data over time as a time series analysis. Other limitations include the lack of use of RSV diagnostic methods in outpatient settings and the use of low-sensitivity diagnostic methods, before the widespread adoption of PCR tests, for the identification of ER and hospitalized RSV cases and failure to test for RSV in all hospitalized cases. The methods to solve these limitations were out of the scope of this study but could be the basis for future research. Despite the limitations surrounding the RSV burden of disease in Mexican children <2yo, the results largely reflected the natural history of RSV throughout the continuum of medical care available in Mexico.

## 6 Conclusions

The results obtained for the period 2011–2019 could be used as a baseline scenario for estimating the seasonal financial impact of RSV. Estimated outpatient visits, ER visits, hospitalizations, length of stay and use of mechanical ventilation can help to calculate the financial impact at national and subnational level. In turn, the financial impact could provide a benchmark for determining the financial/budgetary feasibility of implementing different technologies to prevent severe RSV in ARI and LRTI in the real-life settings of healthcare provided in Mexico to children <2yo prioritizing infants <1yo as this is the group of age most affected. Furthermore, the national results and corresponding state-level findings available in Supplementary Digital Content 8 can serve as a basis for improving operational planning of services during autumn-winter season.

## Data Availability

The original contributions presented in the study are included in the article/Supplementary Material, further inquiries can be directed to the corresponding author.

## References

[1] Centers for Disease Control and Prevention. About RSV. U.S. Available online at: https://www.cdc.gov/rsv/about/index.html (Accessed August 14, 2024).

[2] Benítez-Guerra D Piña-Flores C Zamora-López M Escalante-Padrón F Lima-Rogel V González-Ortiz AM. Respiratory syncytial virus acute respiratory infection-associated hospitalizations in preterm Mexican infants: a cohort study. Influenza Other Respi Viruses. (2020) 14:182–8. 10.1111/irv.12708 PMC7040972 31917902

[3] Viñeta Paramo M Watts AW Bone JN Sadarangani M Bettinger JA Seaton C. RSV Hospital admissions during the first 2 seasons among children with chronic medical conditions. JAMA Netw Open. (2025) 8(7):e2519410. 10.1001/jamanetworkopen.2025.19410 PMC12238895 40627354

[4] Martinón-Torres F Carmo M Platero L Drago G López-Belmonte JL Bangert M. Clinical and economic burden of respiratory syncytial virus in Spanish children: the BARI study. BMC Infect Dis. (2022) 22(1):759. 10.1186/s12879-022-07745-0 PMC9520861 36175846

[5] Li Y Wang X Blau DM Caballero MT Feikin DR Gill CJ. Global, regional, and national disease burden estimates of acute lower respiratory infections due to respiratory syncytial virus in children younger than 5 years in 2019: a systematic analysis. Lancet. (2022) 399(10340):2047–64. 10.1016/S0140-6736(22)00478-0 35598608 PMC7613574

[6] Shi T McAllister DA O'Brien KL Simoes EAF Madhi SA Gessner BD. Global, regional, and national disease burden estimates of acute lower respiratory infections due to respiratory syncytial virus in young children in 2015: a systematic review and modelling study. Lancet. (2017) 390:946–58. 10.1016/S0140-6736(17)30938-8 28689664 PMC5592248

[7] Cai W Tolksdorf K Hirve S Schuler E Zhang W Haas W. Evaluation of using ICD-10 code data for respiratory syncytial virus surveillance. Influenza Other Respi Viruses. (2020) 14(6):630–7. 10.1111/irv.12665 PMC7578302 31206246

[8] Hamilton MA Calzavara A Emerson SD Djebli M Sundaram ME Chan AK. Validating international classification of disease 10th revision algorithms for identifying influenza and respiratory syncytial virus hospitalizations. PLoS One. (2021) 16(1):e0244746. 10.1371/journal.pone.0244746 33411792 PMC7790248

[9] Ortiz-Hernández AA Nishimura KK Noyola DE Moreno-Espinosa S Gamiño A Galindo-Fraga A. Differential risk of hospitalization among single virus infections causing influenza-like illnesses. Influenza Other Respi Viruses. (2019) 13:36–43. 10.1111/irv.12606 PMC6304313 30137695

[10] Vizcarra-Ugalde S Rico-Hernández M Monjarás-Ávila C Bernal-Silva S Garrocho-Rangel ME Ochoa-Pérez UR. Intensive care unit admission and death rates of infants admitted with respiratory syncytial virus lower respiratory tract infection in Mexico. Pediatr Infect Dis J. (2016) 35(11):1199–203. 10.1097/INF.0000000000001262 27276178

[11] Mata-Moreno G Bernal-Silva S García-Sepúlveda CA González-Ortíz AM Ochoa-Pérez UR Medina-Serpa AU. Population-based influenza and respiratory syncytial virus hospitalizations and in-hospital mortality rates among Mexican children less than five years of age. Pediatr Infect Dis J. (2024) 43(6):493–7. 10.1097/INF.0000000000004269 38359346

[12] Gamiño-Arroyo AE Moreno-Espinosa S Llamosas-Gallardo B Ortiz-Hernández AA Guerrero ML Galindo-Fraga A. Epidemiology and clinical characteristics of respiratory syncytial virus infections among children and adults in Mexico. Influenza Other Respi Viruses. (2017) 11(1):48–56. 10.1111/irv.12414 PMC5155644 27439650

[13] Noyola DE Zuviri-González A Castro-García JA Ochoa-Zavala JR. Impact of respiratory syncytial virus on hospital admissions in children younger than 3 years of age. J Infect. (2007) 54:180–4. 10.1016/j.jinf.2006.02.004 16580073

[14] Wong-Chew RM Espinoza MA Taboada B Aponte FE Arias-Ortiz MA Monge-Martínez J. Prevalence of respiratory virus in symptomatic children in private physician office settings in five communities of the state of veracruz, Mexico. BMC Res Notes. (2015) 8:261. 10.1186/s13104-015-1239-0 26108920 PMC4479372

[15] Wong-Chew RM García-León ML Noyola DE Perez Gonzalez LF Gaitan Meza J Vilaseñor-Sierra A. Respiratory viruses detected in Mexican children younger than 5 years old with community-acquired pneumonia: a national multicenter study. Int J Infect Dis. (2017) 62:32–8. 10.1016/j.ijid.2017.06.020 28673837 PMC7110468

[16] Data for Health Initiative. Lineamientos para la elaboración de estudios de carga de enfermedad para el nivel nacional. Bloomberg Philanthropies (2025). Available online at: https://assets.website-files.com/5d7f96ea4cc8598434877fed/5ec2e2bfa3677026804fe353_CargaEnfermedad_v1.pdf (Accessed January 2025).

[17] Secretaría de Salud. Sistema de Urgencias Médicas Hospitalarias (2025). Available online at: http://www.dgis.salud.gob.mx/contenidos/basesdedatos/da_urgencias_gobmx.html (Accessed March 2025).

[18] Secretaría de Salud. Egresos Hospitalarios de la Secretaría de Salud (2025). Available online at: http://www.dgis.salud.gob.mx/contenidos/basesdedatos/da_egresoshosp_gobmx.html (Accessed March 2025).

[19] Secretaría de Salud. Egresos Hospitalarios Sectorial (2025). Available online at: http://www.dgis.salud.gob.mx/contenidos/basesdedatos/da_egresoshosp_gobmx.html (Accessed March 2025).

[20] Instituto Nacional de Estadística y Geografía. Sistema de Estadísticas en Salud en Establecimientos Particulares (2025). Available online at: https://www.inegi.org.mx/programas/salud/ (Accessed April 2025).

[21] Secretaría de Salud. Histórico de bases CLUES (2025). Available online at: http://gobi.salud.gob.mx/Bases_Clues.html (Accessed May 2025).

[22] Higgins JPT Thomas J Chandler J Cumpston M Li T Page MJ. editors. Cochrane handbook for systematic reviews of interventions version 6.4. Cochrane. (2023):736. Available online at: cochrane.org. (Accessed March 25, 2025).

[23] Moola S Munn Z Tufanaru C Aromataris E Sears K Sfetcu R. Systematic reviews of etiology and risk (2020). In: Aromataris E, Lockwood C, Porritt K, Pilla B, Jordan Z, editors. JBI Manual for Evidence Synthesis. JBI. (2024). Available online at: https://synthesismanual.jbi.global. 10.46658/JBIMES-24-06 (Accessed March 25, 2025).

[24] Shamseer L Moher D Clarke M Ghersi D Liberati A Petticrew M. Preferred reporting items for systematic review and meta-analysis protocols (PRISMA-P) 2015: elaboration and explanation. Br Med J. (2015) 350:g7647. 10.1136/bmj.g7647 25555855

[25] Bonita R Beaglehole R Kjellstrõm T. Epidemiología Básica. 2nd ed. Geneva: Organización Mundial de la Salud (2006). Available online at: https://iris.paho.org/handle/10665.2/3153 (Accessed March 25, 2025).

[26] Alba S Verdonck K Lenglet A Rumisha SF Wienia M Teunissen I. Bridging research integrity and global health epidemiology (BRIDGE) statement: guidelines for good epidemiological practice. BMJ Glob Health. (2020) 5:e003236. 10.1136/bmjgh-2020-003236 33115859 PMC7594207

[27] Hoffmann W Latza U Baumeister SE Brünger M Buttmann-Schweiger N Hardt J. Guidelines and recommendations for ensuring good epidemiological practice (GEP): a guideline developed by the German society for epidemiology. Eur J Epidemiol. (2019) 34:301–17. 10.1007/s10654-019-00500-x 30830562 PMC6447506

[28] Consejo Nacional de Población. Conciliación Demográfica 1950 a 2019 y Proyecciones de Población de México 2020 a 2070 (2025). Available online at: https://www.gob.mx/conapo/documentos/bases-de-datos-de-la-conciliacion-demografica-1950-a-2019-y-proyecciones-de-la-poblacion-de-mexico-2020-a-2070 (Accessed April 2025).

[29] Instituto Nacional de Estadística y Geografía. Sistema Nacional de Nacimientos (2025). Available online at: https://www.inegi.org.mx/rnm/index.php/catalog/530 (Accessed January 2025).

[30] Manjarrez ME Rosete DP Rincón M Villalba J Cravioto A Cabrera R. Comparative viral frequency in mexican children under 5 years of age with and without upper respiratory symptoms. J Med Microbiol. (2003) 52:579–83. 10.1099/jmm.0.05007-0 12808080

[31] Catálogo Maestro de Guías de Práctica Clínica: IMSS-376-17. Abordaje diagnóstico terapéutico de la neumonía viral grave. México: Secretaría de Salud; 09/03/2017 Available online at: https://www.imss.gob.mx/sites/all/statics/guiasclinicas/376GER.pdf (Accessed June, 2026)

[32] Caserta MT O’Leary ST Munoz FM Ralston SL O’Leary ST Campbell JD. Ralston SL; committee on infectious diseases. Palivizumab prophylaxis in infants and young children at increased risk of hospitalization for respiratory syncytial virus infection. Pediatrics. (2023) 152(1):e2023061803. 10.1542/peds.2023-061803 37357729

[33] Hammitt LL Dagan R Yuan Y Baca Cots M Bosheva M Madhi SA. Nirsevimab for prevention of RSV in healthy late-preterm and term infants. N Engl J Med. (2022) 386(9):837–46. 10.1056/NEJMoa2110275 35235726

[34] Zar HJ Simões EAF Madhi SA Ramilo O Senders SD Shepard JS. Clesrovimab for prevention of RSV disease in healthy infants. N Engl J Med. (2025) 393(13):1292–303. 10.1056/NEJMoa2502984 40961446

[35] Kampmann B Madhi SA Munjal I Simões EAF Pahud BA Llapur C. Bivalent prefusion F vaccine in pregnancy to prevent RSV illness in infants. N Engl J Med. (2023) 388(16):1451–64. 10.1056/NEJMoa2216480 37018474

