# Supplementary material for: National respiratory syncytial virus acute respiratory infection burden of disease estimate in Mexican children under 2 years
Source: Front Pediatr. 2026 Sep 9;14:1880884. doi: 10.3389/fped.2026.1880884 (PMC13597821; doi:10.3389/fped.2026.1880884)
Supplement: Supplementary file 1 [file Supplementaryfile1.docx]

**National Respiratory Syncytial Virus Acute Respiratory Infection Burden of Disease Estimate in Mexican Children under 2 years.**

**Supplementary Materials**

Supplemental Digital Content 1. **Sources of information used for the population-based RSV BoD analysis**

Table SDC1.1. Medical activity databases used for the analysis

| **Database (Db)** | **Description** | **Observations** |
| --- | --- | --- |
| Módulo de IRAs del *Programa del Niño Sano*. | This Db consolidates basic information for ARI cases in children <5yo treated in public primary care units. Comprises 6 variables in the ARI module. Variables used: Age, state, month. | Public primary care facilities represent on average 58% of all primary care facilities in Mexico. The number of consultancy rooms in such facilities is on average comparable to social security facilities and bigger than for private facilities. On average 10 million ARI outpatient visits in <5yo are registered annually. |
| Urgencias Médicas Hospitalarias | This Db consolidates information for ER visits as reported by public hospitals. Comprises between 25 to 30 variables. Diagnoses are registered by 4-digits ICD-10 codes. Variables used: CLUES, age, state, diagnosis, discharge category, month. | Public hospitals represent on average 17% of all hospitals in Mexico. The number of ER beds in these facilities is on average comparable to social security facilities and bigger than for private facilities. On average 9 million emergency visits in all ages are registered annually. |
| SEHS (Sistema de Egresos hospitalarios sectoriales) | This Db consolidates a selected set of variables for hospitalizations as reported by hospitals of social security institutions. Comprises between 10 to 17 variables. Diagnoses are registered by 4- digits ICD-10 codes. Variables used: CLUES, age, state, diagnosis, discharge category. | Hospitals of social security institutions represent on average 11% of all hospitals in México. The number of hospital beds in these facilities is on average comparable to public facilities and bigger than for private facilities. On average 4 million hospitalizations in all ages are reported annually |
| SAEH (Sistema Automatizado de Egresos Hospitalarios) | This Db consolidates the information for hospitalizations as reported by public hospitals. Diagnoses are registered by 4-digits ICD-10 codes. Comprises between 60 to 90 variables. Variables used: CLUES, age, state, diagnosis, length of stay, use of mechanical ventilation, discharge category, month. | Public hospitals represent on average 17% of all hospitals in Mexico. The number of hospital beds in these facilities is on average comparable to social security facilities and bigger than for private facilities. On average 6 million hospitalizations in all ages are registered annually. |
| SESEP (Sistema de Estadísticas de Salud en Establecimien-tos Particulares) | This Db consolidates the information for hospitalizations as reported by private hospitals. Comprises between 7 to 12 variables. Diagnoses are registered by 3-digits ICD-10 codes. Variables used: CLUES, state, diagnosis, length of stay, discharge category. | Private hospitals represent on average 73% of all hospitals in Mexico. The number of hospital beds in these facilities is on average smaller than those on social security or public facilities. On average 3 million hospitalizations in all ages are registered annually |

Notes: Db: Database. CLUES: Clave Unica de Establecimiento de Salud (ID for healthcare facilites).

Disclaimer: Db consolidate cases. Thus, a potential overlapping of cases between outpatient visits and ER visits can exist. ER Db includes the discharge category Hospitalization, preventing potential overlapping with hospitalization in SAEH Db. Reporting to the different information systems is not legally binding. Thus, a proportion of healthcare facilities might not report per year.

Source: Secretaría de Salud and INEGI (17-22)

Supplemental Digital Content 2. **Number of RSV-cases and correcting underreporting of medical facilities through extrapolation**

The number of RSV-cases in the Db analyzed were obtained by applying equation A (eqA):

Where:

Cx,y are the estimated RSV cases for each age group and year;

ARIx,y,i are acute respiratory infections with selected ICD-10 codes for each age group, year and selected ARI ICD-10 code;

RSVPx,y,i are the proportion of RSV cases in ARI for each i selected ICD-10 code, age group and scenario;

RSVx,y,j are cases reported with specific RSV ICD-10 codes for each age group and year.

x is the age group: <1yo, 1yo for outpatient and ER databases; (<1yo,<=35wga), (<1yo,>35wga), 1yo for hospitalization databases;

i is one of 10 selected ARI ICD-10 codes for ER and hospitalization databases and just one category (ARI) for outpatient databases;

j is one of 3 RSV ICD-10 codes for ER and hospitalization databases with no category for outpatient databases;

y is year for period 2011-2023;

s is the RSV-proportion scenario: Average, Min, Max.

To correct underreporting in the public, social security, and private subsystems, the number of reporting hospitals is compared with the total number of hospitals in each subsystem operating at the state level per year. When a lower number of reporting hospitals is detected relative to the total number of hospitals in operation, the proportionality formula presented with the label eq(B) is applied.

Where

C_ijk_ is the total number of emergency room visits at operating hospitals in the Secretariat of Health subsystem in state i, year j, age k

a_ij_ is the number of emergency room visits reported to the SUMHo in state i for year j, age k

O_ij_ is the total number of operating hospitals in the Ministry of Health subsystem in state i, year j

R_ij_ is the number of hospitals reporting to the SUMHo in state i, year j

i is the set of 32 states

j is the period 2011-2023

k is age 0 years or 1 year

Tables 2.1 and 2.2 present the outpatient and hospital facilities at national level used in the calculations

Table SDC2.1. Outpatient facilities. National level

|  |  | **Social Security** |  |  |  | **Social Security** |  |
| --- | --- | --- | --- | --- | --- | --- | --- |
| **Year** | **Public** |  | **Private** | **Total** | **Public** |  | **Private** |
| 2011 | 17,688 | 5,659 | 3,610 | 26,957 | 66% | 21% | 13% |
| 2012 | 18,025 | 5,707 | 4,433 | 28,165 | 64% | 20% | 16% |
| 2013 | 18,362 | 5,739 | 5,753 | 29,854 | 62% | 19% | 19% |
| 2014 | 18,633 | 5,761 | 6,542 | 30,936 | 60% | 19% | 21% |
| 2015 | 18,951 | 5,794 | 7,182 | 31,927 | 59% | 18% | 22% |
| 2016 | 19,131 | 5,948 | 7,892 | 32,971 | 58% | 18% | 24% |
| 2017 | 19,284 | 6,003 | 8,559 | 33,846 | 57% | 18% | 25% |
| 2018 | 19,536 | 6,029 | 9,141 | 34,706 | 56% | 17% | 26% |
| 2019 | 19,782 | 6,040 | 9,737 | 35,559 | 56% | 17% | 27% |
| 2020 | 19,970 | 6,049 | 10,182 | 36,201 | 55% | 17% | 28% |
| 2021 | 20,119 | 6,052 | 10,778 | 36,949 | 54% | 16% | 29% |
| 2022 | 20,289 | 6,058 | 11,312 | 37,659 | 54% | 16% | 30% |
| 2023 | 20,417 | 6,068 | 11,572 | 38,057 | 54% | 16% | 30% |
| 2011-2023 Average: | | | | | 58% | 18% | 24% |

Source: Registro de CLUES 2024-12.

Table SDC2.2. Hospitals. National level

|  |  | **Social Security** |  |  |  | **Social Security** |  |
| --- | --- | --- | --- | --- | --- | --- | --- |
| **Year** | **Public** |  | **Private** | **Total** | **Public** |  | **Private** |
| 2011 | 633 | 338 | 2,651 | 3,622 | 17% | 9% | 73% |
| 2012 | 659 | 350 | 2,737 | 3,746 | 18% | 9% | 73% |
| 2013 | 671 | 351 | 2,783 | 3,805 | 18% | 9% | 73% |
| 2014 | 687 | 351 | 2,819 | 3,857 | 18% | 9% | 73% |
| 2015 | 703 | 356 | 2,947 | 4,006 | 18% | 9% | 74% |
| 2016 | 723 | 516 | 3,164 | 4,403 | 16% | 12% | 72% |
| 2017 | 738 | 518 | 3,224 | 4,480 | 16% | 12% | 72% |
| 2018 | 752 | 522 | 3,307 | 4,581 | 16% | 11% | 72% |
| 2019 | 763 | 523 | 3,381 | 4,667 | 16% | 11% | 72% |
| 2020 | 786 | 541 | 3,436 | 4,763 | 17% | 11% | 72% |
| 2021 | 791 | 547 | 3,505 | 4,843 | 16% | 11% | 72% |
| 2022 | 799 | 551 | 3,575 | 4,925 | 16% | 11% | 73% |
| 2023 | 805 | 563 | 3,622 | 4,990 | 16% | 11% | 73% |
| 2011-2023 Average: | | | | | 17% | 11% | 73% |

Source: Registro de CLUES 2024-12.

Supplemental Digital Content 3**. Extrapolation**

For the extrapolation of outpatient and emergency consultations, the entire outpatient and hospital infrastructure is considered in the public subsystem, as well as in the social security and private subsystems in each state, applying the Law of Large Numbers. The infrastructure used comes from the CLUE database, presented in Supplemental Digital Content 2. The equation used for the extrapolation is presented with the label Eq(2):

Where

X_ijk_ is the total number of outpatient clinics in state i, year j, age k

a_ij_ is the number of outpatient clinics at the PNS in state i for year j, age k

T_ij_ is the total number of outpatient clinics in state i, year j

U_ij_ is the number of outpatient clinics reporting to the Healthy Child Program in state i, year j

i is the set of 32 states

j is the period 2011-2023

k is age 0 years or 1 year

Supplemental Digital Content 4. Estimation of secondary outcomes

Table SDC4.1. Total RSV cases using Mechanical Ventilation in hospitalization

| **Year** | **Mean** | | | | **Min** | | | | **Max** | | | |
| --- | --- | --- | --- | --- | --- | --- | --- | --- | --- | --- | --- | --- |
|  | **(<1yo,<= 35wga)** | **(<1yo,>35wga)** | **1yo** | **<2yo** | **(<1yo,<=35wga)** | **(<1yo,>35wga)** | **1yo** | **<2yo** | **(<1yo,<=35wga)** | **(<1yo,>35wga)** | **1yo** | **<2yo** |
| 2011 | 377 | 1,052 | 225 | 1,655 | 290 | 810 | 225 | 1,326 | 478 | 1,333 | 225 | 2,036 |
| 2012 | 326 | 908 | 187 | 1,421 | 251 | 699 | 187 | 1,137 | 412 | 1,150 | 187 | 1,750 |
| 2013 | 389 | 1,086 | 199 | 1,675 | 299 | 834 | 199 | 1,332 | 494 | 1,379 | 199 | 2,072 |
| 2014 | 277 | 772 | 165 | 1,214 | 212 | 590 | 165 | 967 | 353 | 984 | 165 | 1,502 |
| 2015 | 372 | 1,039 | 166 | 1,578 | 284 | 791 | 166 | 1,241 | 476 | 1,327 | 166 | 1,969 |
| 2016 | 298 | 831 | 154 | 1,283 | 227 | 632 | 154 | 1,013 | 381 | 1,063 | 154 | 1,599 |
| 2017 | 352 | 983 | 145 | 1,480 | 268 | 748 | 145 | 1,161 | 451 | 1,257 | 145 | 1,852 |
| 2018 | 219 | 612 | 126 | 958 | 165 | 461 | 126 | 753 | 283 | 789 | 126 | 1,197 |
| 2019 | 339 | 946 | 163 | 1,448 | 256 | 714 | 163 | 1,134 | 436 | 1,217 | 163 | 1,817 |
| 2020 | 108 | 302 | 36 | 446 | 81 | 227 | 36 | 345 | 140 | 389 | 36 | 565 |
| 2021 | 80 | 224 | 39 | 343 | 61 | 169 | 39 | 269 | 104 | 289 | 39 | 431 |
| 2022 | 63 | 175 | 134 | 371 | 47 | 130 | 134 | 310 | 81 | 227 | 134 | 443 |
| 2023 | 99 | 277 | 180 | 556 | 74 | 207 | 180 | 461 | 129 | 359 | 180 | 668 |
| 2011-2019 average | 328 | 914 | 170 | 1,412 | 250 | 698 | 170 | 1,118 | 418 | 1,167 | 170 | 1,755 |

Source: Authors’ calculations based upon Db listed in Table SDC1.1 and Table 1 in main text

Table SDC4.2. Total RSV deaths in hospitalization

|  | **Mean** | | | | | **Min** | | | | | **Max** | | | | |
| --- | --- | --- | --- | --- | --- | --- | --- | --- | --- | --- | --- | --- | --- | --- | --- |
| **Year** | **(<1yo,<= 35wga)** | **(<1yo,>35wga)** | **1yo** | **<2yo** | **(<1yo,<=35wga)** | | **(<1yo,>35wga)** | **1yo** | **<2yo** | **(<1yo,<=35wga)** | | **(<1yo,>35wga)** | **1yo** | **<2yo** |  |
| 2011 | 65 | 261 | 97 | 423 | 50 | | 201 | 31 | 282 | 82 | | 330 | 297 | 709 |  |
| 2012 | 56 | 225 | 80 | 361 | 43 | | 173 | 26 | 242 | 71 | | 285 | 245 | 601 |  |
| 2013 | 67 | 269 | 85 | 421 | 52 | | 207 | 27 | 286 | 85 | | 342 | 260 | 687 |  |
| 2014 | 48 | 191 | 71 | 310 | 36 | | 146 | 23 | 205 | 61 | | 244 | 217 | 522 |  |
| 2015 | 64 | 257 | 71 | 392 | 49 | | 196 | 23 | 268 | 82 | | 329 | 217 | 628 |  |
| 2016 | 51 | 206 | 66 | 323 | 39 | | 157 | 21 | 217 | 66 | | 263 | 202 | 531 |  |
| 2017 | 61 | 244 | 62 | 367 | 46 | | 185 | 20 | 251 | 78 | | 311 | 190 | 579 |  |
| 2018 | 38 | 152 | 54 | 244 | 29 | | 114 | 17 | 160 | 49 | | 195 | 165 | 409 |  |
| 2019 | 58 | 234 | 70 | 362 | 44 | | 177 | 23 | 244 | 75 | | 302 | 214 | 591 |  |
| 2020 | 19 | 75 | 16 | 110 | 14 | | 56 | 5 | 75 | 24 | | 96 | 49 | 169 |  |
| 2021 | 14 | 56 | 17 | 87 | 10 | | 42 | 5 | 57 | 18 | | 72 | 52 | 142 |  |
| 2022 | 11 | 43 | 57 | 111 | 8 | | 32 | 18 | 58 | 14 | | 56 | 174 | 244 |  |
| 2023 | 17 | 69 | 77 | 163 | 13 | | 51 | 25 | 89 | 22 | | 89 | 235 | 346 |  |
| 2011-2019 average | 56 | 227 | 73 | 356 | 43 | | 173 | 23 | 239 | 72 | | 289 | 223 | 584 |  |

Source: Authors’ calculations based upon Db listed in Table SDC1.1 and Table 1 in main text

Table SDC4.3. Total RSV days of stay in hospitalization

|  | | **Mean** | | | |  | |  | | **Min** | |  | |  | |  | | **Max** | |  | |  | |  |  |
| --- | --- | --- | --- | --- | --- | --- | --- | --- | --- | --- | --- | --- | --- | --- | --- | --- | --- | --- | --- | --- | --- | --- | --- | --- | --- |
| **Year** | | **(<1yo,<=35wga)** | | **(<1yo,>35wga)** | | **1yo** | | **<2yo** | | **(<1yo,<=35wga)** | | **(<1yo,>35wga)** | | **1yo** | | **<2yo** | | **(<1yo,<=35wga)** | | **(<1yo,> 35wga)** | | **1yo** | | **<2yo** |  |
| 2012 | 18,729 | | 92,750 | | 43,940 | | 155,418 | | 14,419 | | 71,405 | | 43,940 | | 129,763 | | 23,719 | | 117,461 | | 43,940 | | 185,119 | | |
| 2013 | 22,398 | | 110,921 | | 46,832 | | 180,150 | | 17,195 | | 85,154 | | 46,832 | | 149,181 | | 28,429 | | 140,786 | | 46,832 | | 216,046 | | |
| 2014 | 15,928 | | 78,879 | | 38,756 | | 133,563 | | 12,169 | | 60,266 | | 38,756 | | 111,191 | | 20,301 | | 100,538 | | 38,756 | | 159,595 | | |
| 2015 | 21,420 | | 106,075 | | 39,102 | | 166,596 | | 16,313 | | 80,788 | | 39,102 | | 136,203 | | 27,369 | | 135,537 | | 39,102 | | 202,007 | | |
| 2016 | 17,140 | | 84,882 | | 36,234 | | 138,256 | | 13,037 | | 64,565 | | 36,234 | | 113,836 | | 21,925 | | 108,580 | | 36,234 | | 166,739 | | |
| 2017 | 20,269 | | 100,378 | | 33,964 | | 154,612 | | 15,427 | | 76,400 | | 33,964 | | 125,792 | | 25,914 | | 128,331 | | 33,964 | | 188,209 | | |
| 2018 | 12,622 | | 62,506 | | 29,626 | | 104,755 | | 9,517 | | 47,129 | | 29,626 | | 86,272 | | 16,263 | | 80,540 | | 29,626 | | 126,429 | | |
| 2019 | 19,501 | | 96,573 | | 38,396 | | 154,471 | | 14,727 | | 72,932 | | 38,396 | | 126,056 | | 25,094 | | 124,272 | | 38,396 | | 187,763 | | |
| 2020 | 6,219 | | 30,798 | | 8,519 | | 45,536 | | 4,683 | | 23,189 | | 8,519 | | 36,391 | | 8,024 | | 39,735 | | 8,519 | | 56,278 | | |
| 2021 | 4,625 | | 22,906 | | 9,108 | | 36,639 | | 3,490 | | 17,283 | | 9,108 | | 29,881 | | 5,956 | | 29,494 | | 9,108 | | 44,557 | | |
| 2022 | 3,601 | | 17,831 | | 31,417 | | 52,848 | | 2,680 | | 13,272 | | 31,417 | | 47,368 | | 4,688 | | 23,214 | | 31,417 | | 59,319 | | |
| 2023 | 5,704 | | 28,249 | | 42,253 | | 76,206 | | 4,262 | | 21,109 | | 42,253 | | 67,624 | | 7,405 | | 36,670 | | 42,253 | | 86,328 | | |
| 2011-2019 average | | 18,501 | | 91,621 | | 38,356 | | 148,478 | | 14,101 | | 69,830 | | 38,356 | | 122,287 | | 23,627 | | 117,006 | | 38,356 | | 178,988 |  |

Source: Authors’ calculations based upon Db listed in Table SDC1.1 and Table 1 in main text

Supplemental Digital Content 5. Mid-year population per age projected

**Table SDC5.1. Population at risk of developing RSV-related illnesses. National level.**

|  | **Age** | | | | |
| --- | --- | --- | --- | --- | --- |
|  | **(<1yo,<= 35wga)** | **(<1yo,> 35wga)** |  |  |  |
| **Year** |  |  | **<1 yo** | **1 yo** | **< 2 yo** |
| 2011 | 83,726 | 2,181,127 | 2,264,854 | 2,276,358 | 4,541,211 |
| 2012 | 83,324 | 2,154,413 | 2,237,737 | 2,256,378 | 4,494,115 |
| 2013 | 82,588 | 2,130,015 | 2,212,604 | 2,212,604 | 4,425,208 |
| 2014 | 84,187 | 2,108,943 | 2,193,130 | 2,202,204 | 4,395,334 |
| 2015 | 83,484 | 2,086,192 | 2,169,676 | 2,180,861 | 4,350,537 |
| 2016 | 83,712 | 2,049,711 | 2,133,424 | 2,156,706 | 4,290,129 |
| 2017 | 83,491 | 2,012,037 | 2,095,528 | 2,121,365 | 4,216,893 |
| 2018 | 83,484 | 1,995,302 | 2,078,787 | 2,085,059 | 4,163,846 |
| 2019 | 86,007 | 1,989,547 | 2,075,554 | 2,068,964 | 4,144,518 |
| 2020 | 83,228 | 1,986,171 | 2,069,399 | 2,062,364 | 4,131,763 |
| 2021 | 91,706 | 1,965,412 | 2,057,118 | 2,053,110 | 4,110,228 |
| 2022 | 92,944 | 1,939,491 | 2,032,436 | 2,045,477 | 4,077,912 |
| 2023 | 92,500 | 1,913,521 | 2,006,021 | 2,025,380 | 4,031,401 |
| 2011-2019 average: | 83,778 | 2,078,588 | 2,162,366 | 2,173,389 | 4,335,755 |

Source: Authors estimates based upon CONAPO and INEGI (29,30)

**Table SDC5.2. Population at risk of developing RSV-related illnesses. State level. (<1 yo, <=35wga)**

| **State** | **2011** | **2012** | **2013** | **2014** | **2015** | **2016** | **2017** | **2018** | **2019** | **2020** | **2021** | **2022** | **2023** |
| --- | --- | --- | --- | --- | --- | --- | --- | --- | --- | --- | --- | --- | --- |
| Aguascalientes | 956 | 962 | 966 | 997 | 998 | 1,010 | 1,020 | 1,032 | 1,076 | 1,046 | 1,152 | 1,169 | 1,165 |
| Baja California | 2,072 | 2,062 | 2,043 | 2,080 | 2,070 | 2,097 | 2,116 | 2,135 | 2,213 | 2,155 | 2,387 | 2,430 | 2,430 |
| Baja California Sur | 467 | 465 | 460 | 468 | 466 | 471 | 474 | 480 | 502 | 498 | 563 | 580 | 584 |
| Campeche | 607 | 612 | 617 | 640 | 643 | 645 | 635 | 621 | 621 | 583 | 626 | 620 | 604 |
| Coahuila | 2,032 | 2,042 | 2,044 | 2,103 | 2,105 | 2,133 | 2,148 | 2,165 | 2,247 | 2,187 | 2,421 | 2,465 | 2,463 |
| Colima | 457 | 456 | 451 | 457 | 448 | 440 | 432 | 429 | 440 | 423 | 459 | 462 | 459 |
| Chiapas | 4,844 | 4,891 | 4,926 | 5,110 | 5,156 | 5,250 | 5,312 | 5,389 | 5,620 | 5,500 | 6,132 | 6,269 | 6,275 |
| Chihuahua | 2,312 | 2,277 | 2,233 | 2,252 | 2,222 | 2,232 | 2,243 | 2,273 | 2,376 | 2,316 | 2,556 | 2,602 | 2,600 |
| Ciudad de México | 5,094 | 4,990 | 4,863 | 4,863 | 4,696 | 4,586 | 4,473 | 4,345 | 4,319 | 4,045 | 4,389 | 4,425 | 4,372 |
| Durango | 1,334 | 1,332 | 1,326 | 1,358 | 1,353 | 1,357 | 1,345 | 1,330 | 1,348 | 1,274 | 1,368 | 1,360 | 1,333 |
| Guanajuato | 4,290 | 4,279 | 4,246 | 4,336 | 4,311 | 4,343 | 4,364 | 4,404 | 4,574 | 4,434 | 4,862 | 4,893 | 4,827 |
| Guerrero | 2,952 | 2,952 | 2,947 | 3,030 | 3,022 | 3,029 | 3,007 | 2,982 | 3,035 | 2,896 | 3,137 | 3,116 | 3,042 |
| Hidalgo | 1,967 | 1,949 | 1,919 | 1,939 | 1,908 | 1,899 | 1,878 | 1,859 | 1,894 | 1,817 | 1,992 | 2,012 | 2,000 |
| Jalisco | 5,623 | 5,622 | 5,588 | 5,703 | 5,658 | 5,675 | 5,660 | 5,661 | 5,843 | 5,647 | 6,200 | 6,281 | 6,261 |
| México | 11,253 | 11,121 | 10,946 | 11,078 | 10,908 | 10,849 | 10,710 | 10,589 | 10,789 | 10,246 | 11,094 | 11,175 | 11,079 |
| Michoacán | 3,517 | 3,508 | 3,477 | 3,544 | 3,515 | 3,522 | 3,512 | 3,527 | 3,651 | 3,628 | 4,107 | 4,183 | 4,182 |
| Morelos | 1,184 | 1,178 | 1,170 | 1,196 | 1,186 | 1,185 | 1,176 | 1,169 | 1,198 | 1,171 | 1,307 | 1,320 | 1,304 |
| Nayarit | 825 | 819 | 808 | 817 | 805 | 798 | 782 | 771 | 789 | 769 | 856 | 873 | 875 |
| Nuevo León | 3,255 | 3,283 | 3,302 | 3,418 | 3,459 | 3,558 | 3,644 | 3,741 | 3,958 | 3,929 | 4,401 | 4,507 | 4,546 |
| Oaxaca | 3,025 | 2,991 | 2,953 | 3,005 | 2,980 | 2,990 | 2,983 | 2,987 | 3,090 | 3,007 | 3,343 | 3,427 | 3,443 |
| Puebla | 4,812 | 4,770 | 4,719 | 4,811 | 4,782 | 4,818 | 4,836 | 4,878 | 5,090 | 5,008 | 5,622 | 5,801 | 5,861 |
| Querétaro | 1,450 | 1,463 | 1,472 | 1,523 | 1,534 | 1,565 | 1,588 | 1,606 | 1,664 | 1,620 | 1,795 | 1,827 | 1,824 |
| Quintana Roo | 1,013 | 1,026 | 1,033 | 1,069 | 1,085 | 1,126 | 1,168 | 1,217 | 1,306 | 1,307 | 1,466 | 1,494 | 1,490 |
| San Luis Potosí | 1,928 | 1,909 | 1,886 | 1,919 | 1,902 | 1,911 | 1,913 | 1,922 | 1,991 | 1,936 | 2,145 | 2,195 | 2,201 |
| Sinaloa | 1,966 | 1,948 | 1,920 | 1,945 | 1,917 | 1,911 | 1,905 | 1,913 | 1,981 | 1,926 | 2,124 | 2,140 | 2,110 |
| Sonora | 1,827 | 1,816 | 1,797 | 1,825 | 1,800 | 1,791 | 1,770 | 1,758 | 1,808 | 1,758 | 1,948 | 1,981 | 1,978 |
| Tabasco | 1,717 | 1,717 | 1,718 | 1,772 | 1,770 | 1,771 | 1,752 | 1,724 | 1,735 | 1,632 | 1,764 | 1,771 | 1,747 |
| Tamaulipas | 2,226 | 2,201 | 2,167 | 2,196 | 2,168 | 2,173 | 2,174 | 2,182 | 2,262 | 2,203 | 2,430 | 2,455 | 2,438 |
| Tlaxcala | 928 | 926 | 921 | 943 | 941 | 949 | 951 | 958 | 995 | 971 | 1,084 | 1,112 | 1,116 |
| Veracruz | 5,250 | 5,206 | 5,131 | 5,188 | 5,083 | 5,011 | 4,897 | 4,801 | 4,864 | 4,646 | 5,052 | 5,043 | 4,955 |
| Yucatán | 1,313 | 1,321 | 1,324 | 1,364 | 1,367 | 1,386 | 1,397 | 1,409 | 1,462 | 1,421 | 1,558 | 1,558 | 1,530 |
| Zacatecas | 1,232 | 1,227 | 1,216 | 1,238 | 1,227 | 1,228 | 1,224 | 1,225 | 1,266 | 1,230 | 1,365 | 1,399 | 1,407 |

Source: Authors estimates based upon CONAPO and INEGI (29,30)

**Table SDC5.3. Population at risk of developing RSV-related illnesses. State level. (<1 yo, >35wga)**

| **State** | **2011** | **2012** | **2013** | **2014** | **2015** | **2016** | **2017** | **2018** | **2019** | **2020** | **2021** | **2022** | **2023** |
| --- | --- | --- | --- | --- | --- | --- | --- | --- | --- | --- | --- | --- | --- |
| Aguascalientes | 24,899 | 24,884 | 24,914 | 24,978 | 24,944 | 24,740 | 24,576 | 24,654 | 24,895 | 24,971 | 24,691 | 24,402 | 24,098 |
| Baja California | 53,971 | 53,327 | 52,696 | 52,117 | 51,732 | 51,353 | 50,998 | 51,030 | 51,186 | 51,422 | 51,154 | 50,698 | 50,278 |
| Baja California Sur | 12,177 | 12,021 | 11,862 | 11,715 | 11,633 | 11,537 | 11,434 | 11,473 | 11,616 | 11,891 | 12,066 | 12,097 | 12,075 |
| Campeche | 15,818 | 15,822 | 15,907 | 16,035 | 16,063 | 15,782 | 15,300 | 14,854 | 14,369 | 13,902 | 13,415 | 12,943 | 12,498 |
| Coahuila | 52,925 | 52,800 | 52,708 | 52,671 | 52,612 | 52,233 | 51,768 | 51,751 | 51,982 | 52,197 | 51,893 | 51,445 | 50,953 |
| Colima | 11,912 | 11,789 | 11,620 | 11,456 | 11,184 | 10,771 | 10,420 | 10,248 | 10,189 | 10,084 | 9,845 | 9,649 | 9,488 |
| Chiapas | 126,191 | 126,465 | 127,048 | 128,008 | 128,846 | 128,558 | 128,010 | 128,788 | 129,994 | 131,251 | 131,415 | 130,824 | 129,808 |
| Chihuahua | 60,225 | 58,880 | 57,585 | 56,417 | 55,519 | 54,656 | 54,047 | 54,334 | 54,969 | 55,258 | 54,784 | 54,288 | 53,781 |
| Ciudad de México | 132,701 | 129,028 | 125,431 | 121,826 | 117,350 | 112,300 | 107,790 | 103,850 | 99,916 | 96,527 | 94,064 | 92,345 | 90,441 |
| Durango | 34,745 | 34,438 | 34,203 | 34,018 | 33,807 | 33,234 | 32,422 | 31,792 | 31,178 | 30,403 | 29,318 | 28,372 | 27,580 |
| Guanajuato | 111,760 | 110,647 | 109,517 | 108,608 | 107,740 | 106,331 | 105,178 | 105,249 | 105,801 | 105,824 | 104,199 | 102,102 | 99,855 |
| Guerrero | 76,895 | 76,334 | 76,011 | 75,904 | 75,514 | 74,178 | 72,476 | 71,269 | 70,219 | 69,111 | 67,222 | 65,023 | 62,922 |
| Hidalgo | 51,230 | 50,392 | 49,490 | 48,583 | 47,670 | 46,505 | 45,265 | 44,425 | 43,806 | 43,364 | 42,686 | 41,977 | 41,372 |
| Jalisco | 146,491 | 145,363 | 144,107 | 142,857 | 141,387 | 138,952 | 136,405 | 135,297 | 135,156 | 134,767 | 132,872 | 131,060 | 129,520 |
| México | 293,140 | 287,536 | 282,307 | 277,514 | 272,593 | 265,645 | 258,099 | 253,088 | 249,584 | 244,522 | 237,759 | 233,198 | 229,197 |
| Michoacán | 91,630 | 90,694 | 89,678 | 88,776 | 87,838 | 86,239 | 84,635 | 84,306 | 84,454 | 86,579 | 88,030 | 87,282 | 86,515 |
| Morelos | 30,835 | 30,464 | 30,172 | 29,955 | 29,637 | 29,018 | 28,340 | 27,940 | 27,707 | 27,953 | 28,005 | 27,539 | 26,981 |
| Nayarit | 21,486 | 21,188 | 20,842 | 20,468 | 20,107 | 19,546 | 18,853 | 18,429 | 18,247 | 18,343 | 18,348 | 18,207 | 18,101 |
| Nuevo León | 84,793 | 84,885 | 85,162 | 85,627 | 86,429 | 87,110 | 87,816 | 89,413 | 91,550 | 93,759 | 94,321 | 94,049 | 94,044 |
| Oaxaca | 78,791 | 77,343 | 76,155 | 75,265 | 74,472 | 73,210 | 71,883 | 71,397 | 71,485 | 71,760 | 71,653 | 71,521 | 71,221 |
| Puebla | 125,346 | 123,323 | 121,704 | 120,511 | 119,509 | 117,975 | 116,550 | 116,597 | 117,739 | 119,503 | 120,488 | 121,044 | 121,250 |
| Querétaro | 37,763 | 37,819 | 37,955 | 38,157 | 38,333 | 38,319 | 38,262 | 38,377 | 38,491 | 38,652 | 38,464 | 38,126 | 37,736 |
| Quintana Roo | 26,392 | 26,529 | 26,650 | 26,789 | 27,121 | 27,580 | 28,152 | 29,094 | 30,210 | 31,201 | 31,414 | 31,166 | 30,823 |
| San Luis Potosí | 50,223 | 49,365 | 48,641 | 48,066 | 47,529 | 46,790 | 46,102 | 45,925 | 46,067 | 46,189 | 45,976 | 45,809 | 45,528 |
| Sinaloa | 51,222 | 50,376 | 49,514 | 48,722 | 47,897 | 46,791 | 45,897 | 45,712 | 45,818 | 45,969 | 45,531 | 44,660 | 43,639 |
| Sonora | 47,596 | 46,962 | 46,341 | 45,730 | 44,990 | 43,849 | 42,661 | 42,026 | 41,819 | 41,944 | 41,758 | 41,335 | 40,919 |
| Tabasco | 44,740 | 44,398 | 44,297 | 44,396 | 44,237 | 43,375 | 42,211 | 41,210 | 40,135 | 38,952 | 37,798 | 36,947 | 36,141 |
| Tamaulipas | 57,982 | 56,900 | 55,900 | 55,016 | 54,170 | 53,201 | 52,383 | 52,158 | 52,335 | 52,568 | 52,080 | 51,232 | 50,432 |
| Tlaxcala | 24,184 | 23,942 | 23,752 | 23,627 | 23,513 | 23,228 | 22,921 | 22,900 | 23,018 | 23,180 | 23,238 | 23,214 | 23,084 |
| Veracruz | 136,763 | 134,602 | 132,344 | 129,959 | 127,008 | 122,684 | 118,021 | 114,749 | 112,513 | 110,865 | 108,276 | 105,242 | 102,496 |
| Yucatán | 34,205 | 34,164 | 34,147 | 34,164 | 34,158 | 33,947 | 33,675 | 33,679 | 33,809 | 33,905 | 33,385 | 32,503 | 31,648 |
| Zacatecas | 32,098 | 31,734 | 31,356 | 31,010 | 30,651 | 30,076 | 29,488 | 29,288 | 29,293 | 29,353 | 29,262 | 29,195 | 29,097 |

Source: Authors estimates based upon CONAPO and INEGI(29,30)

**Table SDC5.4. Population at risk of developing RSV-related illnesses. State level. (1 yo)**

| **State** | **2011** | **2012** | **2013** | **2014** | **2015** | **2016** | **2017** | **2018** | **2019** | **2020** | **2021** | **2022** | **2023** |
| --- | --- | --- | --- | --- | --- | --- | --- | --- | --- | --- | --- | --- | --- |
| Aguascalientes | 26,029 | 26,066 | 25,880 | 25,993 | 25,986 | 25,970 | 25,865 | 25,737 | 25,880 | 25,828 | 25,521 | 25,416 | 25,202 |
| Baja California | 57,571 | 57,028 | 54,739 | 55,486 | 55,094 | 54,995 | 54,670 | 54,189 | 54,086 | 53,901 | 53,769 | 53,903 | 53,609 |
| Baja California Sur | 12,732 | 12,649 | 12,322 | 12,368 | 12,314 | 12,297 | 12,216 | 12,146 | 12,226 | 12,356 | 12,576 | 12,840 | 12,907 |
| Campeche | 16,504 | 16,573 | 16,524 | 16,729 | 16,768 | 16,596 | 16,174 | 15,641 | 15,159 | 14,829 | 14,506 | 14,101 | 13,656 |
| Coahuila | 55,410 | 55,407 | 54,752 | 54,994 | 54,992 | 55,001 | 54,683 | 54,266 | 54,340 | 54,314 | 54,097 | 54,130 | 53,814 |
| Colima | 12,471 | 12,414 | 12,071 | 12,109 | 11,875 | 11,538 | 11,196 | 10,910 | 10,780 | 10,552 | 10,233 | 10,055 | 9,900 |
| Chiapas | 128,616 | 129,370 | 131,974 | 130,506 | 131,394 | 131,823 | 131,420 | 130,944 | 131,503 | 133,430 | 135,316 | 136,372 | 136,231 |
| Chihuahua | 64,423 | 63,262 | 59,818 | 60,553 | 59,668 | 59,078 | 58,433 | 58,042 | 58,248 | 57,901 | 57,255 | 57,173 | 56,885 |
| Ciudad de México | 137,541 | 134,032 | 130,295 | 125,919 | 121,643 | 117,538 | 113,330 | 108,621 | 104,507 | 100,112 | 96,141 | 94,313 | 92,839 |
| Durango | 36,046 | 35,908 | 35,529 | 35,455 | 35,305 | 34,970 | 34,270 | 33,423 | 32,770 | 32,311 | 31,581 | 30,678 | 29,831 |
| Guanajuato | 116,813 | 115,979 | 113,763 | 113,396 | 112,533 | 111,670 | 110,493 | 109,371 | 109,218 | 109,634 | 109,344 | 108,418 | 106,612 |
| Guerrero | 79,460 | 79,057 | 78,959 | 78,090 | 77,619 | 76,620 | 74,951 | 73,043 | 71,660 | 71,228 | 70,669 | 69,232 | 67,205 |
| Hidalgo | 53,909 | 53,298 | 51,409 | 51,468 | 50,648 | 49,784 | 48,620 | 47,370 | 46,561 | 46,034 | 45,513 | 45,093 | 44,502 |
| Jalisco | 152,492 | 151,795 | 149,695 | 148,796 | 147,459 | 145,861 | 143,488 | 141,062 | 140,186 | 140,082 | 139,237 | 138,251 | 136,844 |
| México | 306,210 | 301,334 | 293,253 | 289,780 | 284,901 | 279,507 | 272,272 | 264,801 | 260,010 | 258,771 | 255,127 | 249,786 | 245,758 |
| Michoacán | 94,743 | 94,202 | 93,155 | 92,215 | 91,400 | 90,318 | 88,805 | 87,581 | 87,217 | 87,307 | 89,425 | 91,530 | 91,105 |
| Morelos | 32,575 | 32,282 | 31,341 | 31,594 | 31,286 | 30,845 | 30,208 | 29,543 | 29,179 | 28,815 | 28,852 | 29,100 | 28,707 |
| Nayarit | 22,457 | 22,315 | 21,650 | 21,718 | 21,425 | 20,991 | 20,345 | 19,752 | 19,473 | 19,397 | 19,487 | 19,618 | 19,529 |
| Nuevo León | 88,822 | 89,131 | 88,464 | 89,393 | 90,196 | 91,362 | 92,135 | 92,831 | 94,450 | 96,196 | 97,770 | 98,951 | 98,934 |
| Oaxaca | 82,134 | 80,987 | 79,108 | 78,628 | 77,883 | 77,075 | 75,852 | 74,673 | 74,370 | 74,322 | 74,186 | 74,592 | 74,713 |
| Puebla | 129,758 | 128,199 | 126,423 | 124,917 | 123,952 | 123,061 | 121,681 | 120,454 | 120,757 | 121,963 | 123,378 | 125,224 | 126,202 |
| Querétaro | 39,136 | 39,291 | 39,426 | 39,419 | 39,608 | 39,826 | 39,825 | 39,611 | 39,595 | 39,820 | 39,990 | 40,050 | 39,849 |
| Quintana Roo | 27,557 | 27,778 | 27,683 | 27,950 | 28,315 | 28,956 | 29,566 | 30,210 | 31,146 | 31,989 | 32,659 | 33,086 | 32,936 |
| San Luis Potosí | 52,680 | 51,919 | 50,527 | 50,268 | 49,709 | 49,217 | 48,535 | 47,856 | 47,697 | 47,734 | 47,603 | 47,702 | 47,710 |
| Sinaloa | 53,613 | 52,979 | 51,434 | 51,254 | 50,495 | 49,660 | 48,764 | 48,041 | 47,816 | 47,645 | 47,447 | 47,293 | 46,537 |
| Sonora | 50,470 | 49,979 | 48,138 | 48,565 | 47,867 | 46,997 | 45,863 | 44,803 | 44,346 | 43,838 | 43,447 | 43,538 | 43,245 |
| Tabasco | 46,830 | 46,636 | 46,015 | 46,394 | 46,236 | 45,641 | 44,566 | 43,246 | 42,097 | 41,054 | 39,857 | 38,938 | 38,196 |
| Tamaulipas | 61,525 | 60,687 | 58,067 | 58,690 | 57,877 | 57,133 | 56,247 | 55,379 | 55,142 | 54,609 | 53,986 | 53,840 | 53,145 |
| Tlaxcala | 25,218 | 25,038 | 24,673 | 24,586 | 24,463 | 24,296 | 23,994 | 23,721 | 23,689 | 23,855 | 24,066 | 24,286 | 24,321 |
| Veracruz | 143,503 | 141,891 | 137,475 | 137,083 | 134,350 | 130,798 | 126,312 | 121,926 | 119,090 | 116,769 | 114,513 | 112,620 | 109,848 |
| Yucatán | 35,899 | 35,901 | 35,471 | 35,637 | 35,649 | 35,702 | 35,542 | 35,299 | 35,348 | 35,371 | 35,222 | 34,907 | 34,116 |
| Zacatecas | 33,208 | 32,991 | 32,572 | 32,253 | 31,954 | 31,581 | 31,046 | 30,567 | 30,420 | 30,398 | 30,336 | 30,441 | 30,491 |

Source: Authors estimates based upon CONAPO and INEGI(29,30)

Supplemental Digital Content 6. RSV-infections seasonality analysis and RSV-cases in ER discharge categories

**SDCT6.1.** Monthly distribution of RSV+ and RSV- among LRTIs ^/1^

|  |  |  |  |  | **% distribution** | | |  |
| --- | --- | --- | --- | --- | --- | --- | --- | --- |
| **Month** | **Total LRTIs** | **RSV -** | **RSV +** | **% RSV +** | | **RSV +** | **RSV -** | |
| January | 806 | 423 | 383 | 47.5 | | 22.1% | 12.7% | |
| February | 682 | 424 | 258 | 37.8 | | 14.9% | 12.7% | |
| March | 553 | 420 | 133 | 24.1 | | 7.7% | 12.6% | |
| April | 278 | 250 | 28 | 10.1 | | 1.6% | 7.5% | |
| May | 140 | 135 | 5 | 3.6 | | 0.3% | 4.0% | |
| June | 100 | 98 | 2 | 2 | | 0.1% | 2.9% | |
| July | 72 | 72 | 0 | 0 | | 0.0% | 2.2% | |
| August | 134 | 125 | 9 | 6.7 | | 0.5% | 3.7% | |
| September | 238 | 210 | 28 | 11.8 | | 1.6% | 6.3% | |
| October | 417 | 304 | 113 | 27.1 | | 6.5% | 9.1% | |
| November | 684 | 401 | 283 | 41.4 | | 16.3% | 12.0% | |
| December | 970 | 477 | 493 | 50.8 | | 28.4% | 14.3% | |
| Total | 5,074 | 3,339 | 1,735 | 34.2 | | 100.0% | 100.0% | |

/1 Data from references 2,9,10,11,13.

**SDCT6.2. RSV cases in ER discharge categories**

| **Year** | **Visit** | **Hospitalization** | **Death** | **Other** |  |
| --- | --- | --- | --- | --- | --- |
| 2011 | 73.83% | 20.18% | 0.07% | 5.93% |  |
| 2012 | 75.83% | 19.88% | 0.07% | 4.23% |  |
| 2013 | 75.54% | 20.20% | 0.06% | 4.21% |  |
| 2014 | 79.07% | 17.17% | 0.05% | 3.72% |  |
| 2015 | 77.97% | 18.40% | 0.05% | 3.58% |  |
| 2016 | 78.98% | 16.98% | 0.08% | 3.96% |  |
| 2017 | 75.20% | 18.61% | 0.08% | 6.11% |  |
| 2018 | 77.43% | 18.28% | 0.09% | 4.20% |  |
| 2019 | 75.87% | 20.22% | 0.06% | 3.85% |  |
| 2020 | 77.05% | 19.83% | 0.18% | 2.95% |  |
| 2021 | 71.80% | 24.72% | 0.14% | 3.34% |  |
| 2022 | 71.73% | 25.52% | 0.10% | 2.64% |  |
| 2023 | 70.75% | 26.89% | 0.10% | 2.26% |  |
| Average 2011-2019 | 75.46% | 20.53% | 0.09% | 3.92% |  |
|  |  |  |  |  |  |

Sources: Authors’ estimates based on descriptive statistical analysis of ER Dbs and RSV cases (2,9,10,11,13)

Supplemental Digital Content 7. **Total RSV cases in outpatient visits, ER visits and hospitalizations**

| **Outpatient visits** | |  |  |  |  |  |  |  |  |  |  |  |  |  |  |
| --- | --- | --- | --- | --- | --- | --- | --- | --- | --- | --- | --- | --- | --- | --- | --- |
|  | **Mean scenario** | |  | **Min scenario** | |  | **Max scenario** | |  | **Mean scenario** | | **Min scenario** | | **Max scenario** | |
| **Year** | **<1 yo** | **1 yo** | **<2yo** | **<1 yo** | **1 yo** | **<2yo** | **<1 yo** | **1 yo** | **<2yo** | **<1 yo** | **1 yo** | **<1 yo** | **1 yo** | **<1 yo** | **1 yo** |
| 2011 | 691,360 | 284,600 | 975,960 | 568,152 | 211,097 | 779,249 | 827,411 | 377,632 | 1,205,043 | 71% | 29% | 73% | 27% | 69% | 31% |
| 2012 | 647,404 | 273,864 | 921,268 | 532,029 | 203,134 | 735,163 | 774,805 | 363,387 | 1,138,192 | 70% | 30% | 72% | 28% | 68% | 32% |
| 2013 | 660,407 | 282,396 | 942,803 | 542,715 | 209,463 | 752,177 | 790,368 | 374,707 | 1,165,075 | 70% | 30% | 72% | 28% | 68% | 32% |
| 2014 | 647,385 | 288,610 | 935,995 | 532,013 | 214,072 | 746,085 | 774,783 | 382,953 | 1,157,736 | 69% | 31% | 71% | 29% | 67% | 33% |
| 2015 | 584,349 | 254,351 | 838,700 | 480,211 | 188,661 | 668,872 | 699,342 | 337,495 | 1,036,837 | 70% | 30% | 72% | 28% | 67% | 33% |
| 2016 | 511,972 | 228,396 | 740,367 | 420,733 | 169,409 | 590,141 | 612,722 | 303,055 | 915,777 | 69% | 31% | 71% | 29% | 67% | 33% |
| 2017 | 420,366 | 187,973 | 608,339 | 345,452 | 139,426 | 484,878 | 503,089 | 249,419 | 752,509 | 69% | 31% | 71% | 29% | 67% | 33% |
| 2018 | 331,894 | 143,575 | 475,469 | 272,747 | 106,495 | 379,241 | 397,207 | 190,508 | 587,715 | 70% | 30% | 72% | 28% | 68% | 32% |
| 2019 | 360,033 | 153,120 | 513,153 | 295,871 | 113,574 | 409,445 | 430,883 | 203,173 | 634,056 | 70% | 30% | 72% | 28% | 68% | 32% |
| 2020 | 137,219 | 57,612 | 194,831 | 112,765 | 42,733 | 155,498 | 164,222 | 76,444 | 240,666 | 70% | 30% | 73% | 27% | 68% | 32% |
| 2021 | 115,257 | 46,158 | 161,416 | 94,717 | 34,237 | 128,954 | 137,938 | 61,247 | 199,185 | 71% | 29% | 73% | 27% | 69% | 31% |
| 2022 | 179,732 | 82,294 | 262,027 | 147,702 | 61,041 | 208,742 | 215,102 | 109,195 | 324,297 | 69% | 31% | 71% | 29% | 66% | 34% |
| 2023 | 187,330 | 77,815 | 265,144 | 153,945 | 57,718 | 211,663 | 224,194 | 103,251 | 327,445 | 71% | 29% | 73% | 27% | 68% | 32% |
| Average 2011-2019 | 539,463 | 232,987 | 772,450 | 443,325 | 172,815 | 616,139 | 645,623 | 309,148 | 954,771 | 70% | 30% | 72% | 28% | 68% | 32% |

| **ER visits** |  |  |  |  |  |  |  |  |  |  |  |  |  |  |  |
| --- | --- | --- | --- | --- | --- | --- | --- | --- | --- | --- | --- | --- | --- | --- | --- |
|  | **Mean scenario** | |  | **Min scenario** | |  | **Max scenario** | |  | **Mean scenario** | | **Min scenario** | | **Max scenario** | |
| **Year** | **<1 yo** | **1 yo** | **<2yo** | **<1 yo** | **1 yo** | **<2yo** | **<1 yo** | **1 yo** | **<2yo** | **<1 yo** | **1 yo** | **<1 yo** | **1 yo** | **<1 yo** | **1 yo** |
| 2011 | 155,654 | 78,274 | 233,928 | 129,159 | 71,202 | 200,360 | 184,625 | 92,842 | 277,467 | 67% | 33% | 62% | 38% | 67% | 33% |
| 2012 | 128,654 | 72,048 | 200,702 | 105,237 | 68,487 | 173,724 | 149,343 | 83,634 | 232,977 | 64% | 36% | 60% | 40% | 64% | 36% |
| 2013 | 143,533 | 73,767 | 217,300 | 117,603 | 69,174 | 186,777 | 168,159 | 86,422 | 254,581 | 66% | 34% | 62% | 38% | 66% | 34% |
| 2014 | 111,725 | 69,716 | 181,442 | 92,708 | 65,178 | 157,886 | 131,998 | 82,366 | 214,363 | 62% | 38% | 57% | 43% | 62% | 38% |
| 2015 | 129,708 | 71,405 | 201,113 | 106,794 | 69,740 | 176,534 | 152,895 | 84,169 | 237,064 | 64% | 36% | 60% | 40% | 64% | 36% |
| 2016 | 116,829 | 67,694 | 184,523 | 89,947 | 65,044 | 154,991 | 128,406 | 74,402 | 202,809 | 63% | 37% | 57% | 43% | 63% | 37% |
| 2017 | 110,899 | 60,287 | 171,186 | 85,733 | 59,598 | 145,332 | 123,149 | 66,946 | 190,095 | 65% | 35% | 59% | 41% | 65% | 35% |
| 2018 | 73,358 | 42,081 | 115,439 | 56,807 | 39,562 | 96,368 | 81,386 | 46,687 | 128,073 | 64% | 36% | 58% | 42% | 64% | 36% |
| 2019 | 107,452 | 54,851 | 162,303 | 82,724 | 52,229 | 134,953 | 120,022 | 61,268 | 181,290 | 66% | 34% | 60% | 40% | 66% | 34% |
| 2020 | 26,358 | 12,772 | 39,129 | 21,027 | 10,609 | 31,636 | 30,433 | 14,746 | 45,180 | 67% | 33% | 63% | 37% | 67% | 33% |
| 2021 | 23,907 | 10,337 | 34,245 | 18,707 | 8,147 | 26,855 | 27,652 | 11,957 | 39,609 | 70% | 30% | 65% | 35% | 70% | 30% |
| 2022 | 50,027 | 22,348 | 72,375 | 38,478 | 20,125 | 58,604 | 56,599 | 25,284 | 81,882 | 69% | 31% | 63% | 37% | 69% | 31% |
| 2023 | 75,739 | 31,827 | 107,566 | 57,473 | 29,255 | 86,728 | 84,961 | 35,702 | 120,663 | 70% | 30% | 65% | 35% | 70% | 30% |
| Average 2011-2019 | 119,757 | 65,569 | 185,326 | 96,301 | 62,246 | 158,547 | 137,776 | 75,415 | 213,191 | 65% | 35% | 59% | 41% | 65% | 35% |

| **Hospitalizations** | |  |  |  |  |  |  |  |  | |  |  |  |  |  |  |
| --- | --- | --- | --- | --- | --- | --- | --- | --- | --- | --- | --- | --- | --- | --- | --- | --- |
|  | **Mean scenario** | | | | **Min scenario** | | | | | **Max scenario** | | | | **Mean/Min/Max scenario** | | |
| **Year** | **<1yo,<=35wga** | **<1yo,> 35wga** | **1 yo** | **<2yo** | **<1yo,<=35wga** | **<1yo,> 35wga)** | **1 yo** | **<2yo** | **<1yo,<=35wga** | | **<1yo,>35wga)** | **1 yo** | **<2yo** | **(<1yo,<=35wga)** | **(<1yo,> 35wga)** | **1 yo** |
| 2011 | 3,082 | 18,625 | 8,943 | 30,650 | 2,373 | 14,338 | 6,885 | 23,596 | 3,904 | | 23,587 | 11,326 | 38,817 | 10% | 61% | 29% |
| 2012 | 2,660 | 16,074 | 8,403 | 27,138 | 2,048 | 12,375 | 6,469 | 20,892 | 3,369 | | 20,357 | 10,641 | 34,368 | 10% | 59% | 31% |
| 2013 | 3,182 | 19,224 | 9,078 | 31,483 | 2,442 | 14,758 | 6,969 | 24,170 | 4,038 | | 24,400 | 11,522 | 39,960 | 10% | 61% | 29% |
| 2014 | 2,262 | 13,671 | 7,164 | 23,097 | 1,729 | 10,445 | 5,473 | 17,647 | 2,884 | | 17,424 | 9,131 | 29,439 | 10% | 59% | 31% |
| 2015 | 3,043 | 18,384 | 8,640 | 30,066 | 2,317 | 14,001 | 6,580 | 22,899 | 3,888 | | 23,490 | 11,039 | 38,417 | 10% | 61% | 29% |
| 2016 | 2,435 | 14,711 | 8,389 | 25,535 | 1,852 | 11,190 | 6,381 | 19,423 | 3,114 | | 18,818 | 10,732 | 32,664 | 10% | 58% | 33% |
| 2017 | 2,879 | 17,397 | 7,750 | 28,025 | 2,191 | 13,241 | 5,898 | 21,331 | 3,681 | | 22,241 | 9,908 | 35,830 | 10% | 62% | 28% |
| 2018 | 1,793 | 10,833 | 5,992 | 18,618 | 1,352 | 8,168 | 4,518 | 14,038 | 2,310 | | 13,958 | 7,721 | 23,990 | 10% | 58% | 32% |
| 2019 | 2,770 | 16,737 | 7,741 | 27,249 | 2,092 | 12,640 | 5,846 | 20,578 | 3,565 | | 21,538 | 9,962 | 35,064 | 10% | 61% | 28% |
| 2020 | 883 | 5,338 | 2,469 | 8,690 | 665 | 4,019 | 1,859 | 6,543 | 1,140 | | 6,887 | 3,185 | 11,211 | 10% | 61% | 28% |
| 2021 | 657 | 3,970 | 2,535 | 7,162 | 496 | 2,995 | 1,913 | 5,404 | 846 | | 5,112 | 3,265 | 9,222 | 9% | 55% | 35% |
| 2022 | 511 | 3,090 | 8,251 | 11,853 | 381 | 2,300 | 6,141 | 8,822 | 666 | | 4,023 | 10,742 | 15,431 | 4% | 26% | 70% |
| 2023 | 810 | 4,896 | 11,669 | 17,375 | 605 | 3,658 | 8,719 | 12,983 | 1,052 | | 6,355 | 15,147 | 22,554 | 5% | 28% | 67% |
| Average 2011-2019 | 2,678 | 16,184 | 8,011 | 26,873 | 2,044 | 12,351 | 6,113 | 20,508 | 3,417 | | 20,646 | 10,220 | 34,283 | 10% | 60% | 30% |

yo: years old. wga: weeks of gestational age at birth. ER: emergency room

Source: Authors estimates based upon (ref ProgNiñoSano,SUMHo,SAEH,SEHS,SESEP,RegCLUES) and PF

Supplemental Digital Content 8. **Incidence rates of RSV-cases per 1,000 children per age group and medical setting.**

**State level.**

**SDCT8.1. RSV cases in ARI for outpatient visits per 1,000 children <1 yo. Mean scenario**

| **State** | **2011** | **2012** | **2013** | **2014** | **2015** | **2016** | **2017** | **2018** | **2019** | **2020** | **2021** | **2022** | **2023** |
| --- | --- | --- | --- | --- | --- | --- | --- | --- | --- | --- | --- | --- | --- |
| Aguascalientes | 310 | 284 | 315 | 270 | 226 | 195 | 178 | 169 | 172 | 61 | 66 | 82 | 90 |
| Baja California | 159 | 140 | 148 | 138 | 128 | 117 | 82 | 74 | 66 | 27 | 20 | 54 | 76 |
| Baja California Sur | 316 | 284 | 283 | 260 | 257 | 246 | 209 | 204 | 152 | 5 | 4 | 6 | 10 |
| Campeche | 510 | 474 | 474 | 436 | 380 | 371 | 281 | 142 | 180 | 69 | 106 | 161 | 220 |
| Coahuila | 286 | 251 | 240 | 200 | 181 | 144 | 45 | 44 | 79 | 169 | 249 | 38 | 25 |
| Colima | 305 | 302 | 293 | 267 | 222 | 202 | 184 | 159 | 165 | 254 | 215 | 76 | 72 |
| Chiapas | 188 | 184 | 162 | 175 | 173 | 143 | 118 | 97 | 114 | 33 | 29 | 88 | 115 |
| Chihuahua | 331 | 341 | 322 | 330 | 280 | 270 | 213 | 166 | 206 | 29 | 28 | 112 | 135 |
| Ciudad de México | 160 | 166 | 192 | 206 | 191 | 170 | 156 | 153 | 166 | 12 | 12 | 96 | 66 |
| Durango | 380 | 357 | 373 | 361 | 365 | 312 | 374 | 136 | 149 | 61 | 56 | 84 | 101 |
| Guanajuato | 444 | 405 | 390 | 365 | 331 | 279 | 223 | 195 | 175 | 63 | 41 | 91 | 102 |
| Guerrero | 324 | 336 | 400 | 379 | 330 | 302 | 218 | 169 | 240 | 40 | 38 | 84 | 89 |
| Hidalgo | 562 | 538 | 590 | 566 | 543 | 506 | 407 | 341 | 374 | 165 | 120 | 174 | 207 |
| Jalisco | 216 | 202 | 197 | 193 | 186 | 169 | 98 | 47 | 64 | 24 | 27 | 38 | 38 |
| México | 264 | 261 | 300 | 315 | 322 | 308 | 266 | 236 | 244 | 98 | 57 | 96 | 95 |
| Michoacán | 293 | 262 | 290 | 272 | 248 | 204 | 202 | 126 | 189 | 80 | 85 | 84 | 36 |
| Morelos | 302 | 296 | 329 | 379 | 379 | 353 | 205 | 186 | 168 | 70 | 68 | 147 | 228 |
| Nayarit | 364 | 352 | 364 | 364 | 309 | 273 | 314 | 70 | 75 | 41 | 67 | 109 | 121 |
| Nuevo León | 192 | 193 | 173 | 176 | 188 | 185 | 179 | 110 | 101 | 42 | 34 | 60 | 88 |
| Oaxaca | 825 | 698 | 649 | 676 | 502 | 340 | 248 | 174 | 215 | 79 | 57 | 155 | 184 |
| Puebla | 297 | 268 | 277 | 274 | 270 | 243 | 217 | 189 | 187 | 65 | 51 | 108 | 91 |
| Querétaro | 347 | 338 | 376 | 351 | 333 | 297 | 281 | 258 | 278 | 123 | 100 | 176 | 126 |
| Quintana Roo | 253 | 241 | 244 | 245 | 236 | 209 | 203 | 188 | 183 | 60 | 63 | 59 | 57 |
| San Luis Potosí | 400 | 364 | 388 | 367 | 350 | 301 | 229 | 214 | 209 | 69 | 22 | 77 | 125 |
| Sinaloa | 368 | 406 | 377 | 369 | 310 | 238 | 203 | 206 | 194 | 86 | 81 | 51 | 141 |
| Sonora | 181 | 189 | 191 | 188 | 178 | 163 | 155 | 123 | 130 | 53 | 60 | 60 | 5 |
| Tabasco | 373 | 310 | 306 | 277 | 248 | 233 | 144 | 37 | 73 | 48 | 58 | 93 | 109 |
| Tamaulipas | 270 | 272 | 293 | 315 | 329 | 263 | 218 | 185 | 187 | 72 | 48 | 49 | 61 |
| Tlaxcala | 483 | 459 | 527 | 485 | 444 | 374 | 217 | 110 | 111 | 49 | 40 | 82 | 121 |
| Veracruz | 184 | 189 | 180 | 164 | 56 | 93 | 131 | 127 | 138 | 56 | 42 | 72 | 83 |
| Yucatán | 387 | 316 | 325 | 333 | 359 | 323 | 308 | 272 | 299 | 112 | 101 | 150 | 74 |
| Zacatecas | 493 | 485 | 506 | 510 | 430 | 391 | 336 | 281 | 305 | 127 | 112 | 125 | 102 |

**SDCT8.2.RSV cases in ARI for outpatient visits per 1,000 children 1 yo. Mean scenario**

| **State** | **2011** | **2012** | **2013** | **2014** | **2015** | **2016** | **2017** | **2018** | **2019** | **2020** | **2021** | **2022** | **2023** |
| --- | --- | --- | --- | --- | --- | --- | --- | --- | --- | --- | --- | --- | --- |
| Aguascalientes | 127 | 119 | 135 | 120 | 98 | 86 | 79 | 73 | 73 | 26 | 27 | 38 | 37 |
| Baja California | 64 | 58 | 63 | 60 | 55 | 51 | 36 | 31 | 28 | 11 | 8 | 25 | 31 |
| Baja California Sur | 129 | 119 | 121 | 114 | 110 | 107 | 91 | 87 | 64 | 2 | 2 | 3 | 4 |
| Campeche | 209 | 199 | 203 | 194 | 165 | 163 | 124 | 61 | 76 | 28 | 41 | 71 | 88 |
| Coahuila | 117 | 105 | 103 | 89 | 78 | 63 | 20 | 19 | 34 | 71 | 100 | 17 | 10 |
| Colima | 124 | 126 | 125 | 117 | 95 | 88 | 80 | 67 | 69 | 106 | 87 | 35 | 30 |
| Chiapas | 79 | 79 | 69 | 80 | 77 | 65 | 54 | 43 | 50 | 14 | 12 | 41 | 48 |
| Chihuahua | 132 | 139 | 138 | 143 | 118 | 116 | 92 | 70 | 86 | 12 | 11 | 51 | 56 |
| Ciudad de México | 66 | 70 | 82 | 92 | 83 | 80 | 69 | 66 | 70 | 5 | 5 | 45 | 28 |
| Durango | 157 | 150 | 159 | 160 | 158 | 137 | 165 | 58 | 63 | 25 | 22 | 37 | 41 |
| Guanajuato | 182 | 170 | 167 | 162 | 143 | 123 | 99 | 84 | 75 | 27 | 16 | 41 | 42 |
| Guerrero | 134 | 143 | 171 | 171 | 145 | 135 | 98 | 74 | 104 | 17 | 15 | 38 | 36 |
| Hidalgo | 228 | 224 | 252 | 248 | 231 | 219 | 176 | 144 | 156 | 68 | 47 | 78 | 84 |
| Jalisco | 89 | 85 | 84 | 86 | 81 | 74 | 43 | 20 | 28 | 10 | 11 | 17 | 15 |
| México | 108 | 110 | 128 | 140 | 139 | 136 | 118 | 102 | 104 | 41 | 22 | 43 | 38 |
| Michoacán | 121 | 111 | 124 | 122 | 108 | 90 | 90 | 55 | 81 | 35 | 35 | 38 | 15 |
| Morelos | 122 | 123 | 141 | 166 | 162 | 154 | 89 | 79 | 71 | 30 | 27 | 67 | 93 |
| Nayarit | 149 | 147 | 156 | 159 | 131 | 118 | 135 | 29 | 31 | 17 | 26 | 48 | 49 |
| Nuevo León | 78 | 81 | 74 | 78 | 81 | 82 | 79 | 48 | 43 | 18 | 14 | 28 | 36 |
| Oaxaca | 338 | 293 | 278 | 300 | 217 | 150 | 110 | 75 | 92 | 33 | 23 | 71 | 77 |
| Puebla | 123 | 113 | 118 | 123 | 118 | 108 | 97 | 82 | 81 | 28 | 21 | 50 | 38 |
| Querétaro | 143 | 143 | 161 | 157 | 146 | 133 | 126 | 113 | 120 | 52 | 40 | 80 | 52 |
| Quintana Roo | 104 | 101 | 104 | 109 | 103 | 92 | 90 | 82 | 79 | 26 | 25 | 26 | 23 |
| San Luis Potosí | 163 | 152 | 166 | 163 | 152 | 133 | 101 | 93 | 90 | 29 | 9 | 35 | 52 |
| Sinaloa | 150 | 169 | 161 | 163 | 133 | 104 | 89 | 89 | 82 | 36 | 33 | 23 | 58 |
| Sonora | 73 | 78 | 81 | 82 | 76 | 70 | 67 | 52 | 55 | 22 | 24 | 27 | 2 |
| Tabasco | 152 | 130 | 131 | 123 | 107 | 103 | 64 | 16 | 31 | 20 | 23 | 42 | 45 |
| Tamaulipas | 109 | 112 | 125 | 137 | 139 | 114 | 95 | 79 | 79 | 30 | 20 | 23 | 25 |
| Tlaxcala | 198 | 193 | 225 | 216 | 193 | 166 | 96 | 48 | 48 | 21 | 16 | 38 | 50 |
| Veracruz | 75 | 79 | 77 | 72 | 24 | 40 | 57 | 54 | 58 | 23 | 17 | 32 | 34 |
| Yucatán | 158 | 132 | 139 | 148 | 156 | 142 | 136 | 117 | 127 | 47 | 40 | 67 | 30 |
| Zacatecas | 204 | 205 | 216 | 227 | 187 | 173 | 149 | 121 | 130 | 54 | 45 | 58 | 42 |

**SDCT8.3.RSV cases in LRTI for ER visits per 1,000 children <1 yo. Mean scenario**

| **State** | **2011** | **2012** | **2013** | **2014** | **2015** | **2016** | **2017** | **2018** | **2019** | **2020** | **2021** | **2022** | **2023** |
| --- | --- | --- | --- | --- | --- | --- | --- | --- | --- | --- | --- | --- | --- |
| Aguascalientes | 32 | 59 | 67 | 50 | 65 | 59 | 69 | 49 | 64 | 18 | 22 | 26 | 70 |
| Baja California | 148 | 145 | 150 | 122 | 108 | 176 | 111 | 124 | 182 | 65 | 27 | 64 | 84 |
| Baja California Sur | 89 | 111 | 90 | 56 | 60 | 65 | 51 | 36 | 39 | 10 | 7 | 12 | 21 |
| Campeche | 36 | 30 | 38 | 27 | 27 | 23 | 12 | 9 | 15 | 3 | 3 | 12 | 18 |
| Coahuila | 140 | 112 | 129 | 97 | 137 | 92 | 86 | 59 | 80 | 14 | 11 | 27 | 56 |
| Colima | 490 | 713 | 822 | 1,255 | 756 | 706 | 296 | 229 | 349 | 110 | 258 | 482 | 695 |
| Chiapas | 2 | 2 | 8 | 15 | 19 | 20 | 9 | 15 | 16 | 5 | 3 | 8 | 12 |
| Chihuahua | 582 | 233 | 110 | 42 | 22 | 15 | 22 | 10 | 16 | 6 | 5 | 14 | 21 |
| Ciudad de México | 47 | 32 | 20 | 27 | 37 | 44 | 65 | 36 | 32 | 12 | 9 | 14 | 31 |
| Durango | 35 | 32 | 36 | 32 | 24 | 22 | 22 | 19 | 20 | 6 | 11 | 23 | 24 |
| Guanajuato | 108 | 111 | 133 | 101 | 137 | 104 | 106 | 73 | 128 | 26 | 33 | 49 | 87 |
| Guerrero | 15 | 9 | 16 | 7 | 10 | 7 | 6 | 2 | 4 | 1 | 2 | 6 | 6 |
| Hidalgo | 51 | 32 | 65 | 40 | 67 | 37 | 80 | 31 | 67 | 12 | 8 | 19 | 32 |
| Jalisco | 54 | 79 | 79 | 48 | 60 | 45 | 41 | 27 | 44 | 9 | 11 | 23 | 32 |
| México | 16 | 13 | 19 | 13 | 20 | 15 | 16 | 13 | 19 | 4 | 4 | 12 | 22 |
| Michoacán | 67 | 60 | 75 | 46 | 78 | 70 | 75 | 27 | 56 | 11 | 11 | 6 | 1 |
| Morelos | 240 | 108 | 159 | 104 | 126 | 144 | 157 | 93 | 128 | 21 | 18 | 71 | 94 |
| Nayarit | 41 | 40 | 54 | 38 | 39 | 29 | 18 | 15 | 28 | 7 | 7 | 20 | 31 |
| Nuevo León | 45 | 49 | 75 | 56 | 59 | 79 | 97 | 50 | 47 | 12 | 9 | 15 | 24 |
| Oaxaca | 15 | 10 | 16 | 13 | 30 | 25 | 19 | 16 | 22 | 4 | 2 | 11 | 14 |
| Puebla | 29 | 22 | 39 | 27 | 36 | 28 | 35 | 22 | 30 | 5 | 4 | 15 | 20 |
| Querétaro | 99 | 104 | 160 | 95 | 145 | 107 | 128 | 71 | 139 | 20 | 12 | 47 | 91 |
| Quintana Roo | 30 | 17 | 32 | 18 | 27 | 28 | 17 | 18 | 28 | 6 | 6 | 10 | 13 |
| San Luis Potosí | 52 | 69 | 67 | 53 | 67 | 73 | 55 | 58 | 66 | 24 | 22 | 29 | 42 |
| Sinaloa | 81 | 107 | 125 | 99 | 121 | 120 | 121 | 68 | 110 | 31 | 17 | 53 | 73 |
| Sonora | 90 | 105 | 106 | 79 | 104 | 121 | 114 | 65 | 81 | 22 | 25 | 36 | 50 |
| Tabasco | 80 | 78 | 104 | 109 | 94 | 83 | 40 | 37 | 74 | 20 | 24 | 46 | 69 |
| Tamaulipas | 80 | 79 | 74 | 72 | 87 | 73 | 84 | 66 | 87 | 26 | 17 | 29 | 38 |
| Tlaxcala | 53 | 40 | 68 | 36 | 57 | 41 | 43 | 29 | 41 | 6 | 6 | 29 | 40 |
| Veracruz | 19 | 14 | 18 | 13 | 23 | 19 | 18 | 13 | 18 | 4 | 3 | 13 | 19 |
| Yucatán | 60 | 37 | 40 | 24 | 34 | 52 | 54 | 29 | 49 | 10 | 13 | 57 | 78 |
| Zacatecas | 63 | 65 | 101 | 79 | 66 | 39 | 47 | 30 | 44 | 16 | 11 | 18 | 32 |

**SDCT8.4.RSV cases in LRTI for ER visits per 1,000 children 1 yo. Mean scenario**

| **State** | **2011** | **2012** | **2013** | **2014** | **2015** | **2016** | **2017** | **2018** | **2019** | **2020** | **2021** | **2022** | **2023** |
| --- | --- | --- | --- | --- | --- | --- | --- | --- | --- | --- | --- | --- | --- |
| Aguascalientes | 16 | 33 | 34 | 31 | 36 | 34 | 37 | 28 | 33 | 9 | 10 | 11 | 30 |
| Baja California | 73 | 79 | 75 | 74 | 58 | 99 | 59 | 70 | 92 | 31 | 12 | 28 | 35 |
| Baja California Sur | 44 | 62 | 45 | 34 | 33 | 37 | 27 | 20 | 20 | 5 | 3 | 5 | 9 |
| Campeche | 18 | 16 | 19 | 17 | 15 | 13 | 6 | 5 | 8 | 1 | 1 | 5 | 7 |
| Coahuila | 70 | 62 | 66 | 60 | 75 | 53 | 46 | 33 | 41 | 7 | 5 | 12 | 23 |
| Colima | 245 | 394 | 416 | 770 | 407 | 398 | 156 | 128 | 176 | 53 | 112 | 217 | 293 |
| Chiapas | 1 | 1 | 4 | 9 | 11 | 12 | 5 | 9 | 9 | 3 | 1 | 3 | 5 |
| Chihuahua | 284 | 126 | 55 | 26 | 12 | 9 | 11 | 5 | 8 | 3 | 2 | 6 | 9 |
| Ciudad de México | 24 | 18 | 10 | 17 | 20 | 25 | 35 | 21 | 16 | 6 | 4 | 6 | 14 |
| Durango | 17 | 18 | 18 | 20 | 13 | 13 | 12 | 11 | 10 | 3 | 5 | 10 | 10 |
| Guanajuato | 54 | 61 | 68 | 63 | 75 | 60 | 57 | 42 | 66 | 13 | 14 | 22 | 36 |
| Guerrero | 7 | 5 | 9 | 4 | 6 | 4 | 3 | 1 | 2 | 0.6 | 0.9 | 3 | 3 |
| Hidalgo | 25 | 17 | 33 | 24 | 36 | 21 | 42 | 18 | 34 | 5 | 4 | 8 | 13 |
| Jalisco | 27 | 44 | 40 | 30 | 33 | 26 | 22 | 16 | 23 | 5 | 5 | 10 | 13 |
| México | 8 | 7 | 10 | 8 | 11 | 9 | 9 | 7 | 10 | 2 | 2 | 5 | 9 |
| Michoacán | 34 | 33 | 39 | 29 | 43 | 40 | 40 | 15 | 29 | 5 | 5 | 3 | 1 |
| Morelos | 119 | 59 | 80 | 64 | 68 | 82 | 83 | 53 | 65 | 10 | 8 | 32 | 39 |
| Nayarit | 21 | 22 | 27 | 23 | 21 | 16 | 9 | 8 | 14 | 3 | 3 | 9 | 12 |
| Nuevo León | 23 | 27 | 38 | 35 | 32 | 46 | 52 | 29 | 24 | 6 | 4 | 7 | 10 |
| Oaxaca | 8 | 6 | 8 | 8 | 17 | 14 | 10 | 9 | 11 | 2 | 1 | 5 | 6 |
| Puebla | 15 | 12 | 20 | 17 | 20 | 16 | 19 | 13 | 16 | 2 | 2 | 7 | 9 |
| Querétaro | 50 | 58 | 82 | 60 | 80 | 62 | 70 | 41 | 72 | 10 | 5 | 21 | 38 |
| Quintana Roo | 15 | 9 | 16 | 11 | 15 | 16 | 9 | 10 | 15 | 3 | 3 | 4 | 5 |
| San Luis Potosí | 26 | 38 | 34 | 33 | 37 | 42 | 30 | 33 | 34 | 12 | 10 | 13 | 17 |
| Sinaloa | 40 | 59 | 63 | 61 | 66 | 68 | 64 | 38 | 56 | 15 | 8 | 23 | 30 |
| Sonora | 45 | 57 | 53 | 48 | 56 | 68 | 60 | 37 | 40 | 11 | 11 | 16 | 21 |
| Tabasco | 40 | 43 | 53 | 67 | 52 | 47 | 22 | 21 | 38 | 9 | 10 | 20 | 29 |
| Tamaulipas | 39 | 43 | 37 | 44 | 47 | 41 | 44 | 37 | 44 | 13 | 7 | 13 | 16 |
| Tlaxcala | 26 | 22 | 35 | 23 | 31 | 24 | 23 | 17 | 21 | 3 | 3 | 13 | 17 |
| Veracruz | 10 | 8 | 9 | 8 | 13 | 11 | 10 | 7 | 9 | 2 | 1 | 6 | 8 |
| Yucatán | 30 | 20 | 20 | 15 | 19 | 30 | 29 | 16 | 25 | 5 | 5 | 25 | 32 |
| Zacatecas | 32 | 36 | 52 | 49 | 36 | 23 | 25 | 17 | 22 | 8 | 5 | 8 | 14 |

**SDCT8.5.RSV cases in LRTI for hospitalizations per 1,000 children (<1yo,<=35wga). Mean scenario**

| **State** | **2011** | **2012** | **2013** | **2014** | **2015** | **2016** | **2017** | **2018** | **2019** | **2020** | **2021** | **2022** | **2023** |
| --- | --- | --- | --- | --- | --- | --- | --- | --- | --- | --- | --- | --- | --- |
| Aguascalientes | 32 | 32 | 36 | 24 | 30 | 24 | 39 | 20 | 26 | 8 | 9 | 3 | 4 |
| Baja California | 48 | 45 | 50 | 38 | 45 | 46 | 42 | 29 | 38 | 18 | 8 | 5 | 7 |
| Baja California Sur | 46 | 52 | 54 | 27 | 36 | 36 | 36 | 24 | 34 | 11 | 7 | 3 | 6 |
| Campeche | 43 | 29 | 37 | 24 | 29 | 19 | 32 | 18 | 21 | 5 | 3 | 3 | 6 |
| Coahuila | 67 | 56 | 68 | 55 | 47 | 44 | 44 | 23 | 33 | 14 | 6 | 5 | 7 |
| Colima | 94 | 54 | 73 | 65 | 90 | 53 | 90 | 46 | 82 | 18 | 24 | 15 | 21 |
| Chiapas | 16 | 17 | 18 | 14 | 14 | 11 | 11 | 8 | 12 | 6 | 6 | 4 | 6 |
| Chihuahua | 58 | 71 | 87 | 73 | 68 | 61 | 68 | 46 | 61 | 39 | 19 | 12 | 18 |
| Ciudad de México | 98 | 91 | 115 | 72 | 129 | 98 | 92 | 69 | 111 | 37 | 20 | 22 | 39 |
| Durango | 57 | 47 | 58 | 47 | 45 | 42 | 47 | 28 | 46 | 16 | 13 | 9 | 12 |
| Guanajuato | 38 | 37 | 44 | 28 | 43 | 30 | 42 | 24 | 41 | 10 | 15 | 6 | 10 |
| Guerrero | 34 | 15 | 24 | 13 | 20 | 14 | 20 | 11 | 19 | 3 | 3 | 3 | 5 |
| Hidalgo | 24 | 16 | 20 | 13 | 23 | 14 | 23 | 11 | 20 | 11 | 2 | 3 | 6 |
| Jalisco | 27 | 23 | 24 | 16 | 24 | 21 | 27 | 15 | 21 | 5 | 6 | 3 | 4 |
| México | 22 | 16 | 19 | 14 | 22 | 16 | 23 | 16 | 23 | 6 | 4 | 3 | 7 |
| Michoacán | 26 | 21 | 27 | 15 | 23 | 16 | 24 | 13 | 25 | 7 | 6 | 5 | 6 |
| Morelos | 34 | 22 | 23 | 15 | 28 | 23 | 27 | 14 | 21 | 5 | 4 | 3 | 4 |
| Nayarit | 37 | 34 | 31 | 26 | 34 | 28 | 28 | 16 | 36 | 16 | 14 | 5 | 9 |
| Nuevo León | 55 | 51 | 57 | 46 | 48 | 37 | 43 | 28 | 35 | 9 | 4 | 4 | 5 |
| Oaxaca | 23 | 18 | 23 | 16 | 21 | 13 | 21 | 10 | 18 | 5 | 2 | 4 | 7 |
| Puebla | 27 | 19 | 28 | 21 | 27 | 20 | 26 | 17 | 27 | 8 | 2 | 4 | 7 |
| Querétaro | 22 | 16 | 26 | 15 | 28 | 17 | 30 | 16 | 29 | 17 | 11 | 10 | 12 |
| Quintana Roo | 28 | 19 | 24 | 16 | 22 | 20 | 26 | 17 | 22 | 8 | 10 | 3 | 4 |
| San Luis Potosí | 40 | 39 | 48 | 31 | 48 | 36 | 38 | 21 | 40 | 11 | 4 | 5 | 8 |
| Sinaloa | 44 | 49 | 58 | 43 | 48 | 46 | 58 | 39 | 49 | 19 | 19 | 7 | 12 |
| Sonora | 56 | 60 | 60 | 39 | 57 | 53 | 45 | 31 | 41 | 12 | 8 | 5 | 6 |
| Tabasco | 44 | 29 | 36 | 26 | 27 | 26 | 35 | 15 | 22 | 5 | 6 | 6 | 8 |
| Tamaulipas | 32 | 30 | 32 | 24 | 31 | 27 | 31 | 21 | 30 | 10 | 5 | 4 | 6 |
| Tlaxcala | 36 | 28 | 39 | 26 | 42 | 23 | 34 | 19 | 36 | 4 | 3 | 8 | 10 |
| Veracruz | 15 | 12 | 14 | 10 | 15 | 13 | 18 | 10 | 14 | 4 | 3 | 2 | 4 |
| Yucatán | 39 | 29 | 42 | 25 | 43 | 45 | 55 | 37 | 34 | 6 | 9 | 12 | 12 |
| Zacatecas | 36 | 42 | 48 | 38 | 37 | 34 | 48 | 21 | 39 | 16 | 9 | 6 | 9 |

**SDCT8.6.RSV cases in LRTI for hospitalizations per 1,000 children (<1yo,>35wga). Mean scenario**

| **State** | **2011** | **2012** | **2013** | **2014** | **2015** | **2016** | **2017** | **2018** | **2019** | **2020** | **2021** | **2022** | **2023** |
| --- | --- | --- | --- | --- | --- | --- | --- | --- | --- | --- | --- | --- | --- |
| Aguascalientes | 8 | 7 | 8 | 6 | 7 | 6 | 10 | 5 | 7 | 2 | 2 | 1 | 1 |
| Baja California | 11 | 11 | 12 | 9 | 11 | 11 | 11 | 7 | 10 | 5 | 2 | 1 | 2 |
| Baja California Sur | 11 | 12 | 13 | 6 | 9 | 9 | 9 | 6 | 9 | 3 | 2 | 1 | 2 |
| Campeche | 10 | 7 | 9 | 6 | 7 | 5 | 8 | 5 | 5 | 1 | 1 | 1 | 2 |
| Coahuila | 15 | 13 | 16 | 13 | 11 | 11 | 11 | 6 | 9 | 4 | 2 | 1 | 2 |
| Colima | 22 | 13 | 17 | 16 | 22 | 13 | 22 | 12 | 21 | 4 | 7 | 4 | 6 |
| Chiapas | 4 | 4 | 4 | 3 | 3 | 3 | 3 | 2 | 3 | 2 | 2 | 1 | 2 |
| Chihuahua | 14 | 16 | 20 | 18 | 16 | 15 | 17 | 12 | 16 | 10 | 5 | 3 | 5 |
| Ciudad de México | 23 | 21 | 27 | 17 | 31 | 24 | 23 | 17 | 29 | 9 | 6 | 6 | 11 |
| Durango | 13 | 11 | 14 | 11 | 11 | 10 | 12 | 7 | 12 | 4 | 4 | 3 | 4 |
| Guanajuato | 9 | 9 | 10 | 7 | 10 | 7 | 11 | 6 | 11 | 3 | 4 | 2 | 3 |
| Guerrero | 8 | 4 | 6 | 3 | 5 | 3 | 5 | 3 | 5 | 1 | 1 | 1 | 1 |
| Hidalgo | 6 | 4 | 5 | 3 | 6 | 3 | 6 | 3 | 5 | 3 | 1 | 1 | 2 |
| Jalisco | 6 | 5 | 6 | 4 | 6 | 5 | 7 | 4 | 6 | 1 | 2 | 1 | 1 |
| México | 5 | 4 | 5 | 3 | 5 | 4 | 6 | 4 | 6 | 1 | 1 | 1 | 2 |
| Michoacán | 6 | 5 | 6 | 4 | 6 | 4 | 6 | 3 | 7 | 2 | 2 | 1 | 2 |
| Morelos | 8 | 5 | 5 | 4 | 7 | 6 | 7 | 4 | 6 | 1 | 1 | 1 | 1 |
| Nayarit | 9 | 8 | 7 | 6 | 8 | 7 | 7 | 4 | 9 | 4 | 4 | 2 | 3 |
| Nuevo León | 13 | 12 | 13 | 11 | 12 | 9 | 11 | 7 | 9 | 2 | 1 | 1 | 2 |
| Oaxaca | 5 | 4 | 5 | 4 | 5 | 3 | 5 | 3 | 5 | 1 | 1 | 1 | 2 |
| Puebla | 6 | 4 | 6 | 5 | 6 | 5 | 6 | 4 | 7 | 2 | 1 | 1 | 2 |
| Querétaro | 5 | 4 | 6 | 4 | 7 | 4 | 7 | 4 | 8 | 4 | 3 | 3 | 4 |
| Quintana Roo | 7 | 4 | 6 | 4 | 5 | 5 | 7 | 4 | 6 | 2 | 3 | 1 | 1 |
| San Luis Potosí | 9 | 9 | 11 | 7 | 12 | 9 | 10 | 5 | 10 | 3 | 1 | 1 | 2 |
| Sinaloa | 10 | 12 | 14 | 10 | 12 | 11 | 14 | 10 | 13 | 5 | 5 | 2 | 4 |
| Sonora | 13 | 14 | 14 | 9 | 14 | 13 | 11 | 8 | 11 | 3 | 2 | 2 | 2 |
| Tabasco | 10 | 7 | 8 | 6 | 7 | 6 | 9 | 4 | 6 | 1 | 2 | 2 | 2 |
| Tamaulipas | 7 | 7 | 8 | 6 | 7 | 7 | 8 | 5 | 8 | 3 | 1 | 1 | 2 |
| Tlaxcala | 8 | 6 | 9 | 6 | 10 | 6 | 8 | 5 | 9 | 1 | 1 | 2 | 3 |
| Veracruz | 4 | 3 | 3 | 2 | 4 | 3 | 4 | 2 | 4 | 1 | 1 | 1 | 1 |
| Yucatán | 9 | 7 | 10 | 6 | 10 | 11 | 14 | 9 | 9 | 2 | 3 | 3 | 4 |
| Zacatecas | 8 | 10 | 11 | 9 | 9 | 8 | 12 | 5 | 10 | 4 | 2 | 2 | 3 |

**SDCT8.7.RSV cases in LRTI for hospitalizations per 1,000 children 1yo. Mean scenario**

| **State** | **2011** | **2012** | **2013** | **2014** | **2015** | **2016** | **2017** | **2018** | **2019** | **2020** | **2021** | **2022** | **2023** |
| --- | --- | --- | --- | --- | --- | --- | --- | --- | --- | --- | --- | --- | --- |
| Aguascalientes | 3 | 4 | 4 | 3 | 3 | 3 | 4 | 3 | 3 | 1 | 2 | 2 | 3 |
| Baja California | 5 | 5 | 5 | 4 | 5 | 6 | 4 | 4 | 4 | 2 | 1 | 3 | 5 |
| Baja California Sur | 5 | 6 | 6 | 3 | 4 | 5 | 4 | 3 | 4 | 1 | 1 | 2 | 4 |
| Campeche | 5 | 3 | 4 | 3 | 3 | 3 | 3 | 2 | 2 | 0 | 0 | 2 | 4 |
| Coahuila | 7 | 7 | 7 | 7 | 5 | 6 | 5 | 3 | 4 | 2 | 1 | 3 | 4 |
| Colima | 10 | 6 | 8 | 8 | 10 | 7 | 9 | 6 | 9 | 2 | 4 | 11 | 14 |
| Chiapas | 2 | 2 | 2 | 2 | 2 | 2 | 1 | 1 | 1 | 1 | 1 | 3 | 4 |
| Chihuahua | 6 | 8 | 9 | 9 | 7 | 8 | 7 | 6 | 7 | 4 | 3 | 9 | 12 |
| Ciudad de México | 11 | 11 | 12 | 9 | 14 | 13 | 10 | 9 | 13 | 4 | 3 | 17 | 26 |
| Durango | 6 | 5 | 6 | 6 | 5 | 6 | 5 | 4 | 5 | 2 | 2 | 6 | 8 |
| Guanajuato | 4 | 4 | 5 | 3 | 5 | 4 | 4 | 3 | 5 | 1 | 3 | 5 | 7 |
| Guerrero | 4 | 2 | 3 | 2 | 2 | 2 | 2 | 1 | 2 | 0 | 1 | 2 | 3 |
| Hidalgo | 3 | 2 | 2 | 2 | 2 | 2 | 2 | 1 | 2 | 1 | 0 | 2 | 4 |
| Jalisco | 3 | 3 | 3 | 2 | 3 | 3 | 3 | 2 | 2 | 1 | 1 | 2 | 3 |
| México | 2 | 2 | 2 | 2 | 2 | 2 | 2 | 2 | 3 | 1 | 1 | 2 | 4 |
| Michoacán | 3 | 2 | 3 | 2 | 3 | 2 | 3 | 2 | 3 | 1 | 1 | 4 | 4 |
| Morelos | 4 | 2 | 2 | 2 | 3 | 3 | 3 | 2 | 2 | 1 | 1 | 2 | 2 |
| Nayarit | 4 | 4 | 3 | 3 | 4 | 4 | 3 | 2 | 4 | 2 | 2 | 4 | 6 |
| Nuevo León | 6 | 6 | 6 | 6 | 5 | 5 | 5 | 4 | 4 | 1 | 1 | 3 | 4 |
| Oaxaca | 3 | 2 | 2 | 2 | 2 | 2 | 2 | 1 | 2 | 1 | 0 | 3 | 4 |
| Puebla | 3 | 2 | 3 | 3 | 3 | 3 | 3 | 2 | 3 | 1 | 0 | 3 | 5 |
| Querétaro | 2 | 2 | 3 | 2 | 3 | 2 | 3 | 2 | 3 | 2 | 2 | 7 | 8 |
| Quintana Roo | 3 | 2 | 3 | 2 | 2 | 3 | 3 | 2 | 3 | 1 | 2 | 2 | 3 |
| San Luis Potosí | 4 | 4 | 5 | 4 | 5 | 5 | 4 | 3 | 5 | 1 | 1 | 4 | 6 |
| Sinaloa | 5 | 6 | 6 | 5 | 5 | 6 | 6 | 5 | 6 | 2 | 3 | 5 | 8 |
| Sonora | 6 | 7 | 6 | 5 | 6 | 7 | 5 | 4 | 5 | 1 | 1 | 4 | 4 |
| Tabasco | 5 | 3 | 4 | 3 | 3 | 3 | 4 | 2 | 3 | 1 | 1 | 4 | 5 |
| Tamaulipas | 3 | 3 | 3 | 3 | 3 | 4 | 3 | 3 | 3 | 1 | 1 | 3 | 4 |
| Tlaxcala | 4 | 3 | 4 | 3 | 5 | 3 | 4 | 3 | 4 | 0 | 0 | 6 | 7 |
| Veracruz | 2 | 1 | 2 | 1 | 2 | 2 | 2 | 1 | 2 | 0 | 1 | 2 | 3 |
| Yucatán | 4 | 3 | 4 | 3 | 5 | 6 | 6 | 5 | 4 | 1 | 2 | 9 | 8 |
| Zacatecas | 4 | 5 | 5 | 5 | 4 | 5 | 5 | 3 | 5 | 2 | 1 | 4 | 6 |
